# Structural and mechanistic insights into Quinolone Synthase to address its functional promiscuity

**DOI:** 10.1038/s42003-024-06152-2

**Published:** 2024-05-14

**Authors:** Mallika Vijayanathan, Abhinav Koyamangalath Vadakkepat, Kozhinjampara R. Mahendran, Abdoallah Sharaf, Kristian E. H. Frandsen, Debashree Bandyopadhyay, M. Radhakrishna Pillai, Eppurath Vasudevan Soniya

**Affiliations:** 1 Transdisciplinary Research Program, Rajiv Gandhi Centre for Biotechnology, Thiruvananthapuram, 695014 India; 2 Molecular Biophysics Unit, Indian Institute of Science, Bangalore, India; 3 SequAna Core Facility, Department of Biology, University of Konstanz, Konstanz, Germany; 4 Genetic Department, Faculty of Agriculture, Ain Shams University, Cairo, 11241 Egypt; 5 Department of Biological Sciences, Birla Institute of Technology and Science, Hyderabad, India; 6 Cancer Research Program, Rajiv Gandhi Centre for Biotechnology, Thiruvananthapuram, 695014 India; 7 Present Address: Department of Plant and Environment Sciences, University of Copenhagen, 1871 Frederiksberg C, Denmark; 8 Present Address: Department of Molecular and Cell Biology, University of Leicester, Henry Wellcome Building, Lancaster Road, Leicester, LE17HB UK

**Keywords:** X-ray crystallography, Biotechnology

## Abstract

Quinolone synthase from *Aegle marmelos* (AmQNS) is a type III polyketide synthase that yields therapeutically effective quinolone and acridone compounds. Addressing the structural and molecular underpinnings of AmQNS and its substrate interaction in terms of its high selectivity and specificity can aid in the development of numerous novel compounds. This paper presents a high-resolution AmQNS crystal structure and explains its mechanistic role in synthetic selectivity. Additionally, we provide a model framework to comprehend structural constraints on ketide insertion and postulate that AmQNS’s steric and electrostatic selectivity plays a role in its ability to bind to various core substrates, resulting in its synthetic diversity. AmQNS prefers quinolone synthesis and can accommodate large substrates because of its wide active site entrance. However, our research suggests that acridone is exclusively synthesized in the presence of high malonyl-CoA concentrations. Potential implications of functionally relevant residue mutations were also investigated, which will assist in harnessing the benefits of mutations for targeted polyketide production. The pharmaceutical industry stands to gain from these findings as they expand the pool of potential drug candidates, and these methodologies can also be applied to additional promising enzymes.

## Abstract (web summary)

A high-resolution structure provides a mechanistic explanation to the synthetic selectivity of quinolone synthase (AmQNS) and possible consequences of functionally relevant residue mutations.

## Introduction

Polyketides (PKs) are chemically diverse natural products with immense pharmaceutical properties^1^. PKs and their possible derivatives could be used as attractive starting points for the development of new bioactive molecules with clinical applications^2,3^. Polyketide synthases (PKS) are multifunctional enzymes that synthesize PKs in plants, fungi, and bacteria^4^. PKS machinery is an ideal target for synthesizing a wide range of architecturally diverse natural products through protein engineering and combinatorial biosynthesis^5^, because of its unique features such as i) wide substrate affinity, ii) alternating condensation steps, and iii) generation of diverge cyclic intermediates^6^. There are three distinct forms of PKS^1^, specifically type I, II, and III, which are categorized based on their protein architecture and reaction mechanism. Type III PKSs, in contrast to type I and type II enzymes, are homodimers and have a relatively smaller size. They facilitate polyketide formation by sequentially adding ‘malonate building blocks’ to a starter substrate (acyl thioester)^7,8^. Each functional unit of type III enzyme contains two ketosynthase (KS) domains (∼40–45 kDa, ~350-390 amino acids per monomeric unit)^9–11^. Type III PKSs are further categorized into two subtypes, viz, the i) chalcone-forming (chalcone synthase (CHS) and ii) non-chalcone-forming (non-CHS), based on the reaction they catalyze^12,13^.

Quinolone synthase (AmQNS)^14^ from the Indian bael tree (*Aegle marmelos* (L.) Correa. or *Crateva marmelos*; common names *-* stone apple or wood apple) belongs to the non-CHS group of type III PKS. The natural substrate for AmQNS is *N*-methyl anthraniloyl-CoA, and the main metabolites are quinolones and acridones. Anthranilic acid-derived quinolone alkaloids (quinine, chloroquine, etc.) have been previously reported to possess antibacterial, anticancer, and antiviral properties^15,16^ and these compounds could serve as potential pharmacological leads for the development of novel drugs. The fundamental mechanism catalyzed by AmQNS yields diketide 4-hydroxy 1-methyl 2-quinolone (89%) via a single-step condensation reaction between *N*-methyl anthraniloyl-CoA and malonyl-CoA. Acridone (11%) is synthesized in a three-step condensation process that begins with the same substrate and employs the same enzyme^14^. When *P*-coumaroyl-CoA is employed as the starting substrate, AmQNS can also produce benzalacetone^14^. Mori et al.^17^ thoroughly investigated the structure and activity of two AmQNS-homolog type-III PKSs found in *Citrus microcarpa*, namely acridone synthase (CmACS) and quinolone synthase (CmQNS). These enzymes also utilize *N*-methyl anthraniloyl-CoA as their initial substrate. Despite the considerable sequence and structural similarities between AmQNS, CmACS, and CmQNS, their product formation patterns, and catalytic efficiencies are substantially different^14^. i.e., In contrast to AmQNS, which yields both acridone and quinolone, CmQNS produces 4-hydroxy-*N*-methylquinolone as a “single product” through the one-step condensation of malonyl-CoA and *N*-methylanthraniloyl-CoA, while CmACS yields both acridone and a variety of other products. Specifically, CmQNS synthesizes the quinolone scaffold by the use of a considerably smaller active site cavity than CmACS, whereas CmACS uses an active site cavity similar to those of CHS to generate acridone^17^. A key determinant of the enzyme’s preference for particular substrates is the residue substitutions in the functionally relevant region. Therefore, even a minor amino acid substitution can have a considerable effect on the functionality of an enzyme. It is remarkable to note that AmQNS and its nearest homolog CmACS both have distinct amino acid variations that favor interaction with the bulky *N*-methyl anthraniloyl-CoA, while hindering the binding of small substrate CoAs. Hence, it is imperative to comprehend the evolutionary, structural, and functional characteristics, along with the reaction mechanism, of these enzymes. Having this information will assist in investigating their potential to produce primary chemical scaffolds to accelerate the process of natural product discovery through metabolite engineering.

Here we present the first-ever high-resolution crystal structures of AmQNS in both an apo form and in complex with substrate and decipher its synthetic selection at the structural and molecular levels. We used semi-empirical quantum chemistry molecular simulations to identify rate-limiting reaction steps leading to the formation of quinolone and acridone scaffolds. Additionally, we have conducted quantum chemical transition state calculations to compare the relative kinetic barriers and thermodynamic enthalpies of substrates. These calculations have provided clear evidence that AmQNS exhibits a structural preference for the quinolone production. Ultimately, the initial evolutionary studies, along with the subsequent structural findings and simulation-based reaction studies, uncover the mechanistic behavior of AmQNS. This knowledge will eventually assist to engineer and repurpose the enzymatic reaction to expand the natural product reservoir for bioprospecting and drug discovery in the future.

## Results and discussion

### Sequence diversity of Type III PKSs and evolutionary position of AmQNS

**Fig. 1:**
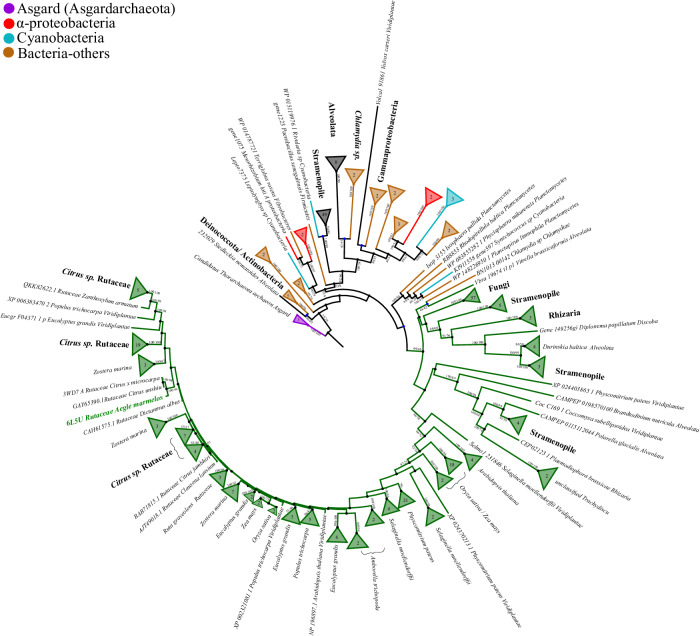
Maximum likelihood (ML) phylogenetic tree of type III PKS protein homologs. This rooted phylogenetic tree illustrates potential evolutionary relationships between identified prokaryotic and eukaryotic homologs (During the homology search, out of the 283 screened species, type III PKS homologs were identified only in 112 species). In the phylogenetic tree, most sequences were collapsed for simplicity and AmQNS’s (6L5U) phylogenetic position is highlighted in green font. We searched for type III PKS in prokaryotes including the Archaeal-Asgard group as well and found homologs in one Asgard species too (*Candidatus Thorarchaeota archaeon*) (details in Supplementary Data 1–4). The Asgard (or Asgardarchaeota- superphylum consisting of a group of archaea) group is a distinct domain of life that represents eukaryotes’ closest prokaryotic relatives^86,87^. These findings suggest that the Asgardarchaeota group may have been the emergence point of the type III PKS enzyme in the tree of life. Hence, the tree was rooted using Asgard homologs to assess the evolutionary direction of the proteins. Moreover, the identified HGT nodes were labeled in blue circles while the phylogeny tree crown (-where all eukaryotic proteins were clusterred) was labeled in dark green. The maximum likelihood branch support values are represented by percentages (calculated in IQ-TREE/RAxML-NG).

The evolutionary investigation of AmQNS, along with its potential homologs from other genera and species (Supplementary Data 1–4), demonstrated a high degree of sequence conservation. The phylogenetic tree in Fig. 1 demonstrates AmQNS’s evolutionary placement, indicating that it is highly conserved and clustered with other Rutacean family members.

We conducted a thorough phylogenetic study to investigate the evolutionary diversity of AmQNS homologs (both putative and verified) across various taxa and to analyze the evolutionary route that contributes to biodiversity. Our evolutionary analysis revealed six events of horizontal gene transfer (HGT), with four of them taking place within the bacterial domain and close to the root of the tree (Fig. 1). The first HGT was identified in a position very near to the root of the tree, between *Siedleckia nematoides* (a parasitic Apicomplexa that belongs to alveolates and infect marine invertebrates) and homologs of Actinobacteria and Deinococcota. The second HGT was identified between most stramenopiles (Heterokonts) and the cyanobacterial Rivularia homolog. Hence, these stramenopile homologs have a plastidial origin. The third HGT event was noted between most alveolates and two of Chlamydia sp. homologs. A fourth HGT was found between the homologs of Gammaproteobacteria and the single Viridiplantae homolog of *Volvox carteri* (a colonial green algae; an excellent model for investigating evolutionary processes^18,19^) in the bacterial domain (Fig. 1). The fourth and fifth occurrences of horizontal gene transfer (HGT) (considered to be the most recent) occurred within the eukaryotic domain (the second major cluster depicted in Fig. 1, and this domain includes several bacterial groups positioned adjacent to the core eukaryotic cluster, behaving like as an outgroup). So specifically, the fifth HGT was found between the homologs of Chlamydia and *Vitrella brassicaformis* (a unicellular photosynthetic alga belongs to Chromerida, a phylum of unicellular alveolates); forming a sister group with the dinoflagellate *Durinskia baltica* (belongs to Alveolata; found in freshwater/brackish/marine environments), *Diplonema papillatum* (heterotrophic marine microeukaryotes belongs to Discoba), all fungi, Rhizaria, and some homologs of Stramenopiles. Finally, the sixth HGT was observed between all the Planctomycetes and cyanobacterial Synechococcus sp. homologs, and all the eukaryotic- homologs in the crown of the tree (Fig. 1; phylogenetic tree crown depicted in dark green).

**Fig. 2:**
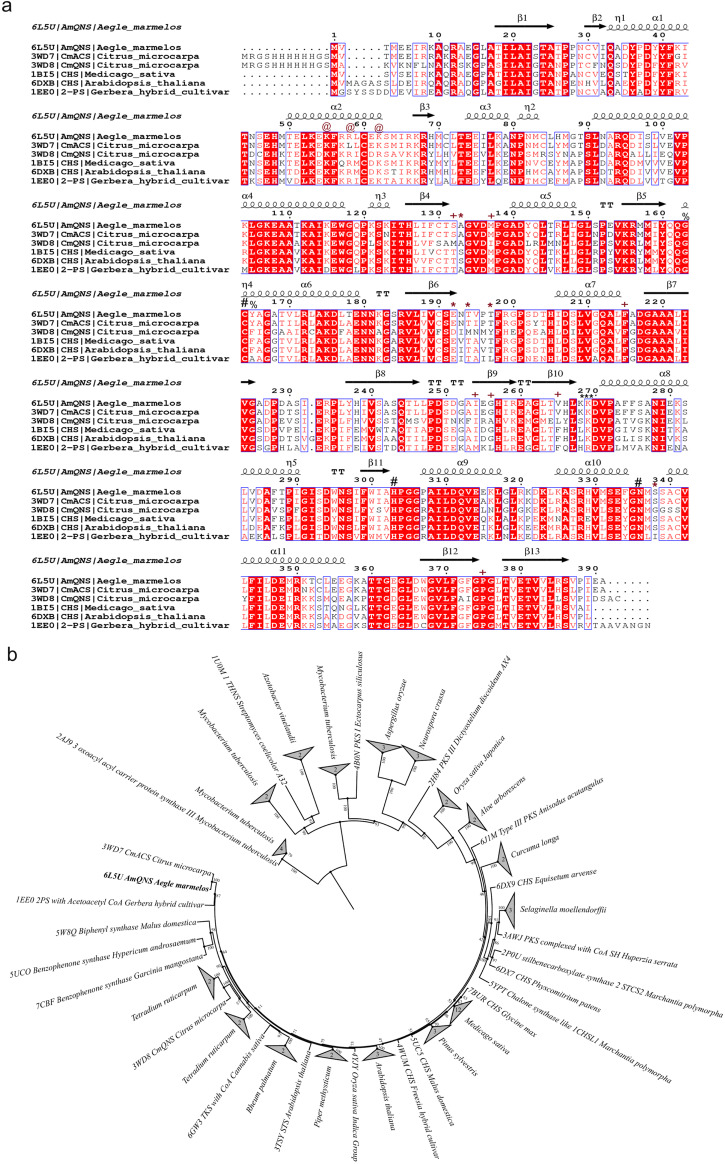
Sequence-structure alignment between different homologs and their evolutionary positions. **a** Alignment was prepared using ClustalW^73^ and visualized in ESPript 3.0^74^. The PDB IDs are used to represent the sequences. The conservation level is shown by a color gradient (white-poor conservation, red-high conservation). Functionally important residues are highlighted ('**#**'—catalytic residues, '* in red'—residues in the substrate-binding pocket, '**+**'—residues in the cyclization pocket), '@'—residues in the CoA-binding tunnel, '%'—residues adjacent to catalytic C164, '* in black'—β-turn region of AmQNS. **b** ML phylogenetic tree showing the evolutionary relationship among the structural homologs in RCSB PDB. Position of AmQNS is highlighted in bold.

Therefore, our phylogenetic analysis provides evidence for the bacterial ancestry of this group of enzymes and indicates that it likely emerged in the early stages, possibly even existing in the Last Eukaryote Common Ancestor (LECA). Throughout the evolutionary route, AmQNS, which descended from a common ancestor, certainly underwent multiple occurrences of horizontal gene transfers (HGT). Besides, there were subsequent occurrences of gene duplication within the Viridiplantae domain. Furthermore, when considering the conservation pattern, a structure-based sequence alignment of AmQNS with its adjacent homologs [from RCSB Protein Data Bank (PDB)] demonstrated a high level of sequence conservation and functional conservancy (Fig. 2a). In addition, the ML phylogenetic tree (Fig. 2b) illustrates the evolutionary connections between the AmQNS structural homologs available in RCSB PDB. The tree clearly indicates that CmACS has the highest degree of structural similarity. However, minor amino acid differences, particularly in the CoA binding/substrate binding/cyclization pocket area, were observed which have a major impact on substrate specificity and/or selectivity, which could contribute to diverse product profile formation via various protein-ligand interactions.

### Catalytic site flexibility facilitates AmQNS substrate promiscuity

One of the most essential aspects of enzymes that determines their unique reaction is the specific interaction between proteins and ligands (such as substrates or cofactors). Our prior radio-TLC experiments have demonstrated that AmQNS has the capacity to accept numerous starting CoAs as possible substrates in vitro^14^. Moreover, in silico studies also indicate that non-physiological substrates could be employed as potential AmQNS ligands^20^. The binding mechanism of several acyl-CoA substrates (small aliphatic to bulky aromatic) with AmQNS was further validated using Surface Plasmon Resonance (SPR) based assays, which enable for real-time monitoring of kinetic parameters^21^. The high affinity and reasonable interaction between the AmQNS and small molecule ligands are indicated by the K_D_ values, which varied from nanomolar to micromolar range (2 nM-2.8 μM). AmQNS demonstrated a high affinity for *N*-methylanthraniloyl-CoA, feruloyl-CoA, and hexanoyl-CoA (with *K*_D_ of 2.04 nM, 9.83 nM, and 7.30 nM, respectively), and it is worth noting that AmQNS prefers bulkier substrates than short acyl- CoAs (Supplementary Fig. 1). The affinity characteristics were consistent with our previously reported interaction studies using thin-layer chromatography (TLC)^14^, and when comparing the steady-state kinetic parameters for AmQNS with different starter substrate CoAs, it is notable that K_m_ values are higher than *K*_D_ for the majority of the substrates (for *N*-methylanthraniloyl CoA-2.93 μM; *p*-coumaroyl CoA-3.62 μM; Feruloyl CoA-9.14 μM). This suggests that catalysis is more rapid than dissociation. These findings imply the prospect of utilizing various substrates to create novel chemical scaffolds, and the enzyme can be further engineered to accommodate various substrates to boost the catalytic versatility.

### Unveiling the complexity: high-resolution AmQNS X-ray crystal structures

**Fig. 3:**
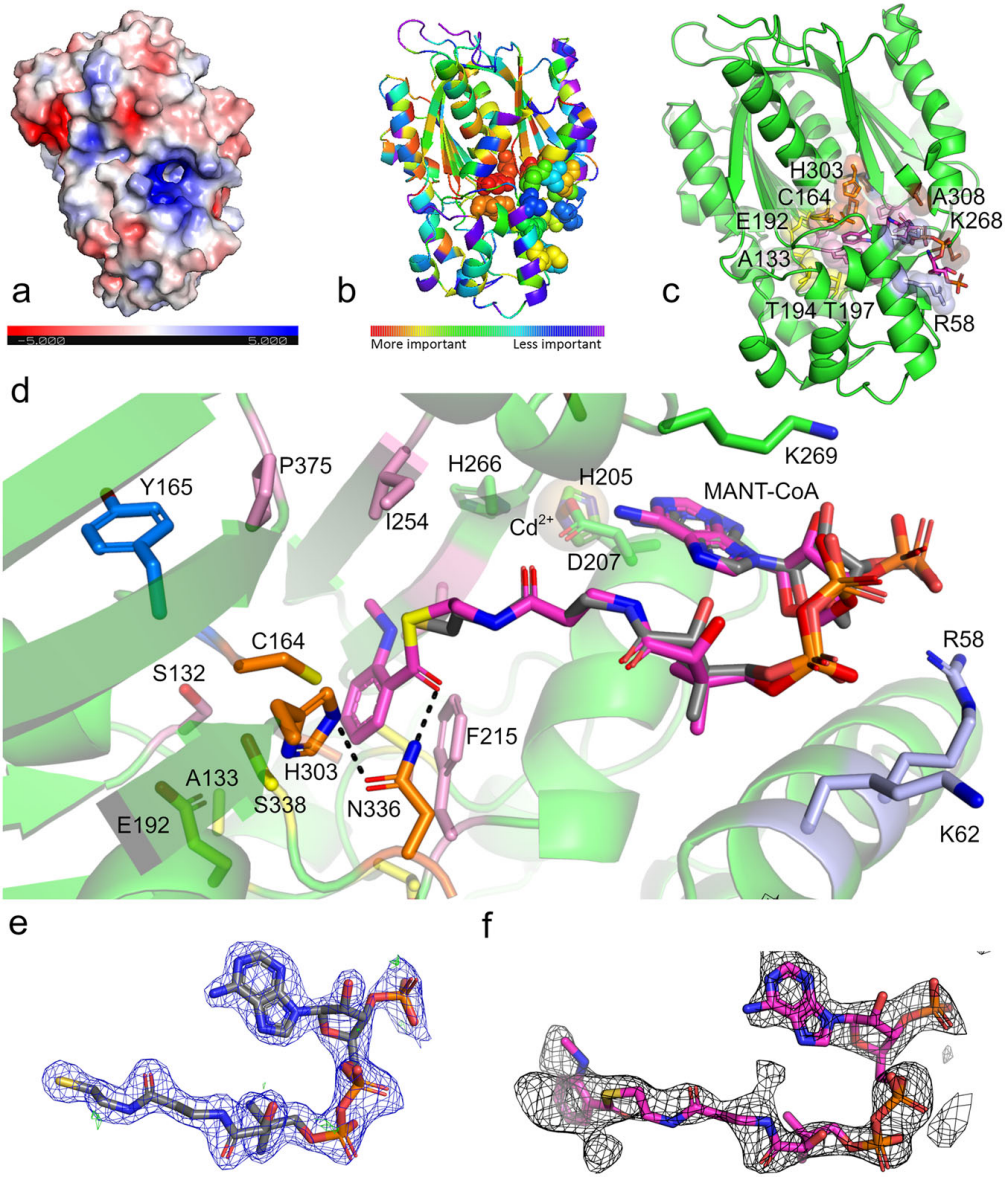
The structure of AmQNS in native apo and substrate-bound form. **a** AmQNS native structure (surface representation, PDB ID: 6L5U, Resolution 1.85 Å) displaying electrostatic charge distribution (positively charged residues in blue and negatively charged residues in red. **b** Evolutionary trace on native AmQNS showing functionally relevant residue positions in the structure. **c** AmQNS—substrate-bound form (cartoon and surface representation, PDB ID: 7CCT, Resolution 2.35 Å). The substrate *N*-methylanthraniloyl CoA (MANT-CoA) is shown in magenta. The following functionally important residues are highlighted in stick and colored spheres: catalytic triad (C164, H303, N336)—in orange, substrate-binding residues (A133, E192, T194, T197, S338)—in yellow, residues in cyclization pocket (S132, M137, F215, I254, G256, V265, P375)—in pink, residues in CoA-binding tunnel (K55, R58, K62)—in light blue, residues adjacent to the catalytic C164 (G163, Y165)—in marine blue; and other residues that form polar contacts with substrate (K268, A308) in brown. **d** Substrate binding environment of MANT-CoA (PDB: 7CCT)—enlarged view. MANT-CoA is shown in magenta and the CoASH ligand (from PDB 6L7J) is superimposed onto the structure for comparison (gray). The cadmium ion is shown as a transparent sphere and the metal interacting residues, H205, D207 and H266, in green sticks. **e** Electron density 2F_o_-F_c_ (blue) and F_o_-F_c_ (green) maps of the CoASH ligand (PDB 6L7J) contoured at 1.0 and 3.0 sigma, respectively. **f** Unbiased polder map calculated around the substrate MANT-CoA (contoured at 3.0 sigma).

The AmQNS crystals provided high-resolution structures with well-defined electron density maps. These structures have been deposited (PDB IDs: 6L5U, 6L7J and 7CCT) in the RCSB Protein Data Bank^22^. Details on crystallization, data collection, and refinement statistics are given in Supplementary Table 1 and Table 1, respectively. The elucidated AmQNS native apo structure has structural folds that are comparable to other type III PKSs (Fig. 3a–c). This structure also displays a conserved topology, which contains a specific upper domain ‘αβαβα’ (ketosynthase domain)^23^. This topology is conserved in all structural homologs, and the lower domain of the protein contains most of the residues that are responsible for binding the substrate (A133, E192, T194, T197, S338; inferred from previously published type III PKS structures and literature references^20^). In the AmQNS monomer, these domains are made up of three β sheets (13 strands (22.4%), 16 α-helices (37.6%), 3–10 helices (2.7%), and other secondary structure elements (37.3%- including four β hairpins, four β bulges, 30 β turns, two ɣ turns) (Fig. 3b, c, Supplementary Fig. 2). The AmQNS protein is functionally active in dimeric form, but each monomer is related by crystallographic symmetry in the crystals leading to one molecule per asymmetric unit (1 mol/ASU). In each monomer, the β-sheets are organized into two antiparallel β-sheets and one mixed sheet, where the strands are arranged in the AmQNS structure’s core, whereas the α-helices are distributed on the surface. The prospective substrate-binding pocket entrance of each AmQNS monomeric unit is bordered by the side chains of the α-helices and β-strands.

**Table 1:** Data collection and refinement statistics (molecular replacement)

	AmQNS Native apo	AmQNS-CoASH bound	AmQNS-MANT-CoA bound
**PDB accession**	6L5U	6L7J	7CCT
**Data collection**			
Space group	H32	H32	H32
Number of molecules/ASU	1	1	1
Cell dimensions			
* a*, *b*, *c* (Å)	149.84, 149.84, 105.491	150.86, 150.86, 105.61	148.839, 148.839, 105.34
α, β, γ (°)	90, 90, 120	90, 90, 120	90, 90, 120
Resolution (Å)	40.93-1.85 (1.95-1.85)^a^	35.23-1.80 (1.92-1.80)	48.76 - 2.35 (2.434 - 2.35)
*R*_sym_ or *R*_merge (%)_	13.3 (100)	14.0(91.7)	14.0(85.8)
*I* / σ*I*	9.9 (1.79)	14.0 (3.42)	8.5(2.00)
Completeness (%)	99.03 (100)	97.60 (99.69)	96.23 (99.25)
Redundancy	11 (10.8)	11.2(11.3)	10.5(10.6)
CC1/2 (%)	99.7	99.7	99.0
**Refinement**			
Resolution (Å)	31.83-1.85	27.38 – 1.80	48.76 – 2.35
No. reflections	38375 (3842)	41643 (4211)	18064 (1844)
*R*_work_ / *R*_free_	0.185/0.220	0.175/ 0.199	0.231/ 0.292
No. atoms	3118	3220	2991
Protein	2872	2282	2879
Ligand/ion	9	48	59
Water	237	290	38
*B*-factors	38.86	33.23	61.25
Protein	37.79	32.19	60.58
Ligand/ion	69.81	50.33	97.19
Water	47.81	40.79	56.81
R.m.s. deviations			
Bond lengths (Å)	0.007	0.007	0.008
Bond angles (°)	0.888	1.31	1.08

^a^Values in parentheses are for the highest-resolution shell.

The orientation and locations of the catalytic residues in AmQNS are comparable to those in the closest homologs (Supplementary Fig. 3, 4). Despite being structurally comparable even at the active site entrance, AmQNS has a considerably bigger binding pocket than its nearest functional homologs from *Citrus X Microcarpa* (PDB IDs: 3WD7 & 3WD8) (Supplementary Fig. 5). This could be the consequence of amino acid substitutions (e.g. F265V, where the smaller valine (V) frees up more space) in the AmQNS active site pocket and the longer tunnel enables the entry of bulky substrates (e.g., *N*-methylanthraniloyl-CoA). The substrate-binding residues in CmACS and AmQNS are essentially identical. Nonetheless, CmQNS showed modest variations, as demonstrated by changes in the cavity volume metrics (reduced parameters) (Supplementary Fig. 5), and even though these sequence alterations and their orientations are minimal, this can lead to potential differences in pocket volume and a shift in the product formation profile.

AmQNS prefers bulkier substrates and the electrostatic potential surface calculation revealed that the substrate-binding pocket regions of AmQNS have a predominantly positive charge (Fig. 3a), which facilitates binding with the phosphate groups of the preferred starter substrate CoAs. These positions of positive charges are consistently found across type III PKSs as binding of the CoA portion of substrates is conserved. Both hydrogen bonds (~32), and nonbonded interactions (~318) make up the AmQNS dimeric interface area (2463–2481 Å). In addition, six prospective salt bridges participating in stabilizing the protein-protein interface [residues involved - D96, D136, D251, H257, R259, K281 (chain A) and R259, H257, R146, D136, D96, E153 (chain B)]. The catalytic triad (C164-H303-N336) located in the upper domain are deeply embedded within the entrance cavity with orientation and position strikingly similar to those of the homologs^17^. In addition, the amino acid residue cysteine C164 in the catalytic triad is a strong nucleophile^24^ (reflected in its pKa value and reactivity of its thiol group) and plays a key role in facilitating thioester exchange events^25^. The reduction of the sulfur donor molecule in enzyme catalysis is very important since it binds to the substrate. The catalytic C164 in AmQNS shows higher nucleophilicity and is more vulnerable to oxidation, consistent with previous research on euphyllophyte CHSs^26,27^. Indeed, crystallographic data of both the native apo- and the CoASH bound form of AmQNS (PDB 6L5U and 6L7J, respectively) supports C164 in an oxidized form with a S-sulfinylation modification (Supplementary Fig. 6a, b). The electron density map of MANT-CoA bound AmQNS did not convincingly support a C164 modification. Furthermore, the electron density of the CoASH-bound AmQNS shows modified C71, which can be accounted for an S-sulfenylation modification (Supplementary Fig. 6c). These observed modifications are in full agreement with predictions made by the pCysMod server^28^ (Supplementary Table 2). This finding aligns with prior research on type III PKS (particularly CHSs), which indicates that CHSs in euphyllophytes have an oxidized form of the catalytic cysteine, while basal plant forms (lycophyte and a moss species) preserve the catalytic cysteine in a reduced state^27^. Interestingly, molecular evolution also plays a crucial role in maintaining the active-site environment of type III PKS proteins. According to Liou et al.^27^, CHSs from basal land plants have fewer reactive catalytic cysteines than CHSs from higher plants. It is unclear whether these findings regarding the modulation of catalytic cysteine reactivity represent a general pattern in non-chalcone-forming PKS family members as well. However, AmQNS has a highly nucleophilic cysteine (C164) in the catalytic region, indicating that it might have evolved to have a high catalytic potential.

The amino acid residues K269, A308, and N336 form polar contacts (2.2–2.9 Å) with the CoA molecule in substrate-bound AmQNS (Supplementary Fig. 7). The interaction with *N*-methyl anthraniloyl CoA (MANT-CoA) was also confirmed by 76 non-bonded interactions. One of them is F215 which appears to be involved in aromatic π-π stacking interaction with the *N-*methylanthraniloyl part of the MANT-CoA ligand (Fig. 3d). An unbiased polder map around the substrate MANT-CoA (contoured at 3.0 sigma) is shown in Fig. 3f. Likewise, K55, L267, G305, and A308 establish hydrogen bonds (distances of 2.5-3.1 Å) in the CoASH bound form (PDB ID: 6L7J, Supplementary Fig. 8). K55 is located in the CoA-binding tunnel at the entrance, and G305 has previously been reported to play a role in shaping the appropriate geometry of the active site pocket^29^. Figure 3e shows the CoASH ligand’s electron density (2Fo-Fc and Fo-Fc maps, contoured at 1.0 and 3.0 sigma). Additionally, thermal disorder parameters might indicate conformational flexibility^30^, and we observed that ligand binding causes well-defined conformational changes in proteins, particularly in the β-turn region of AmQNS (residues K268-K269-D270). All aligned proteins exhibit complete conservation of D270, while K269 is mostly conserved with the exception of 2PS from Gerbera, which has K269R alteration (Fig. 2a). However, only AmQNS and CmACS maintain the K268. CmQNS has a K268S substitution, while other homologs have either ‘K268L’ or ‘K268H’. Comparison studies indicated conformational flexibility at the substrate-binding pocket entrance in AmQNS, which suggested hinge-like movement of the K268-K269-D270 surface loop. Interestingly, in the vicinity of K268-K269-D270, we observed the presence of a large peak in F_o_-F_c_ electron density difference map. This was best modeled as a cadmium ion (from crystallization conditions) interacting H205, D207 and H266 in configuration (geometry, ligand contacts and vacancy) in agreement with CheckMyMetal server^31^ (Fig. 3d). Moreover, this conformational flexibility in the AmQNS enzyme structure provides a larger passageway for a substrate to enter the internal active binding site, which is more evident from the following simulation experiments.

### Structural basis for AmQNS synthetic selectivity

**Fig. 4:**
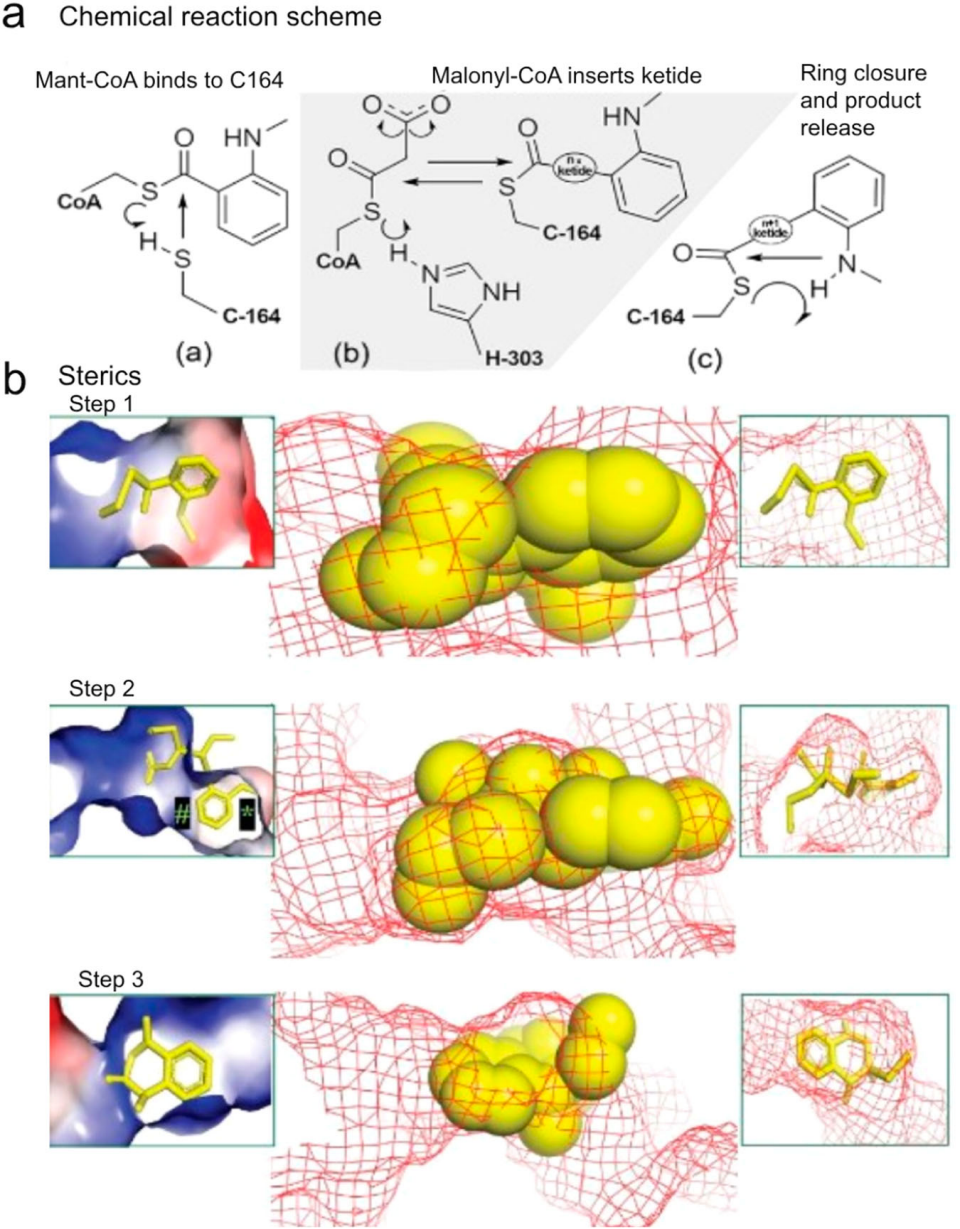
Three steps for AmQNS catalysis. **a** Chemical reaction scheme showing the substrate binding to the enzyme and subsequent product formation. **b** Molecular models show different transition state for complexation between the substrate and enzyme. The effect of specific structural features (steric and electrostatics) on kinetic properties for the subsequent reaction (ketide insertion) is represented in the molecular model (‘#‘ and ‘*‘ markings convey that the receptor poses negligible clashes with the substrate during the first ketide insertion. Still, it should be noted that there is no considerable excess of space available at positions ‘#‘ and ‘*‘ in the first insertion. Consequently, during the process of second and third ketide insertions, during which the reaction intermediate is growing, clashes would be expected at both positions ‘#‘ and ‘*.’). Transition steps can be better viewed in Supplementary Movies (1–3).

To gain insights into the reaction mechanism, followed by the structural elucidation, molecular simulation studies were used to investigate the mechanistic basis of AmQNS synthetic selectivity. Here we examined if specific ligand-protein interactions can be mapped to characterize the enzyme’s relative propensity to select an optimal number of intermediate ketide insertions. We calculated transition states for MANT-CoA binding to AmQNS and defined the three reaction steps (Fig. 4) required for AmQNS-driven quinolone production. The first step entails a classic SN2 thiol addition^32,33^, through which the MANT-CoA substrate binds to the catalytic C164. The second reaction depicts a ketide unit’s concerted process from malonyl-CoA inserts between the cysteine sulfur and the carbonyl carbon of the substrate enzyme complex. The third reaction is then a reverse substitution through which the substrate amine induces product ring closure, which restores the enzymatic cysteine (Fig. 4a). The chemical structure of MANT-CoA, its derivatives and products (quinolone/acridones) are given in Supplementary Fig. 9. The activation energy and enthalpy for each step of the reaction process are provided in Supplementary Table 3.

The multiple transition states^34–37^ for initial complexation between the substrate and enzyme were then demonstrated (Fig. 4b). Transition states tend to be the portion of any reactive progression, where structural features have the greatest impact on the kinetic properties for the subsequent reaction. In the particular case of MANT-CoA binding to the AmQNS active site, it is apparent that the substrate is a good fit for the enzyme, as there are minimal clashes that could either kinetically disfavor or completely abrogate the subsequent reaction. Several observations are made regarding areas on the substrate for which clash is very minimal that (theoretically) increased substrate bulk might reduce the activation barrier (i.e., improve reaction kinetics) through favorable van der Waals (vdW) or electrostatic interactions^38–40^. The observation that the receptor is spacious around the aminomethyl substrate group led to the notion of experimentally investigating whether (computationally) the aminomethyl group could be productively modified as a chloro analog (somewhat bulkier in a potentially favorable manner). Notably, the red and blue receptor patches in the MANT aryl ring region are similarly motivated to explore if a slightly more polar version of the substrate (with a pyridinyl ring, rather than benzyl) might produce kinetically favorable electrostatic complementarity.

Notably, step 2 in the reaction sterics (Fig. 4b) represents prospective receptor structural influences on the first ketide insertion kinetics. In this case, the transition state for this reaction is well accommodated by the receptor, which, in turn, corroborates the prior observation that AmQNS is a viable ‘enzymatic engine’ for promoting quinolone synthesis. Importantly, we propose that second and third ketide insertions may be somewhat less favored than the first insertion. Nonetheless, during the first insertion, there is no considerable excess of space available. As a result, clashes would be expected (in places ‘#‘ and ‘*‘) during the second and third ketide insertions, when the reaction intermediate is growing. These clashes may be somewhat defused with a ligand conformational shift that orients the ring slightly out of the plane of this graphic as the aryl ring begins to progress toward the narrow product exit channel, whose position is relatively well marked in the figure (‘*‘). In step 3, we see transitional interactions between the forming quinolone product and the receptor. It is interesting to note that although the receptor is not hugely antagonistic to product formation, it also does not seem ideally suited, as apparent in the steric clash between the enzymatic surface and the aminomethyl. This clash might be alleviated through a change of conformational twist (to reorient the aryl ring) that is essentially the same factor identified earlier in reaction step 2 as a requisite step for second or third ketide insertions. This has an exciting implication and, this means that although the analysis of step 2 has pointed firmly toward smaller quinolone product formation (compared to a larger acridone product), a kinetic hitch in the final step of quinolone formation may nullify this difference. In Supplementary Table 3, we show the computed impacts of the two minor (chloro and pyridinyl) modifications to the MANT-CoA substrate. Our data show that the substrate modifications appear to have only minor influence, and it is difficult to predict if either shift will produce a demonstrable improvement in reactive profile relative to unmodified MANT-CoA. Alternatively, we also suggest that AmQNS may support a variety of analogs to the standard biologically processed substrates, meaning that their synthetic chemistry can be extended from the production of novel natural product scaffolds to a related display chemical analog.

**Fig. 5:**
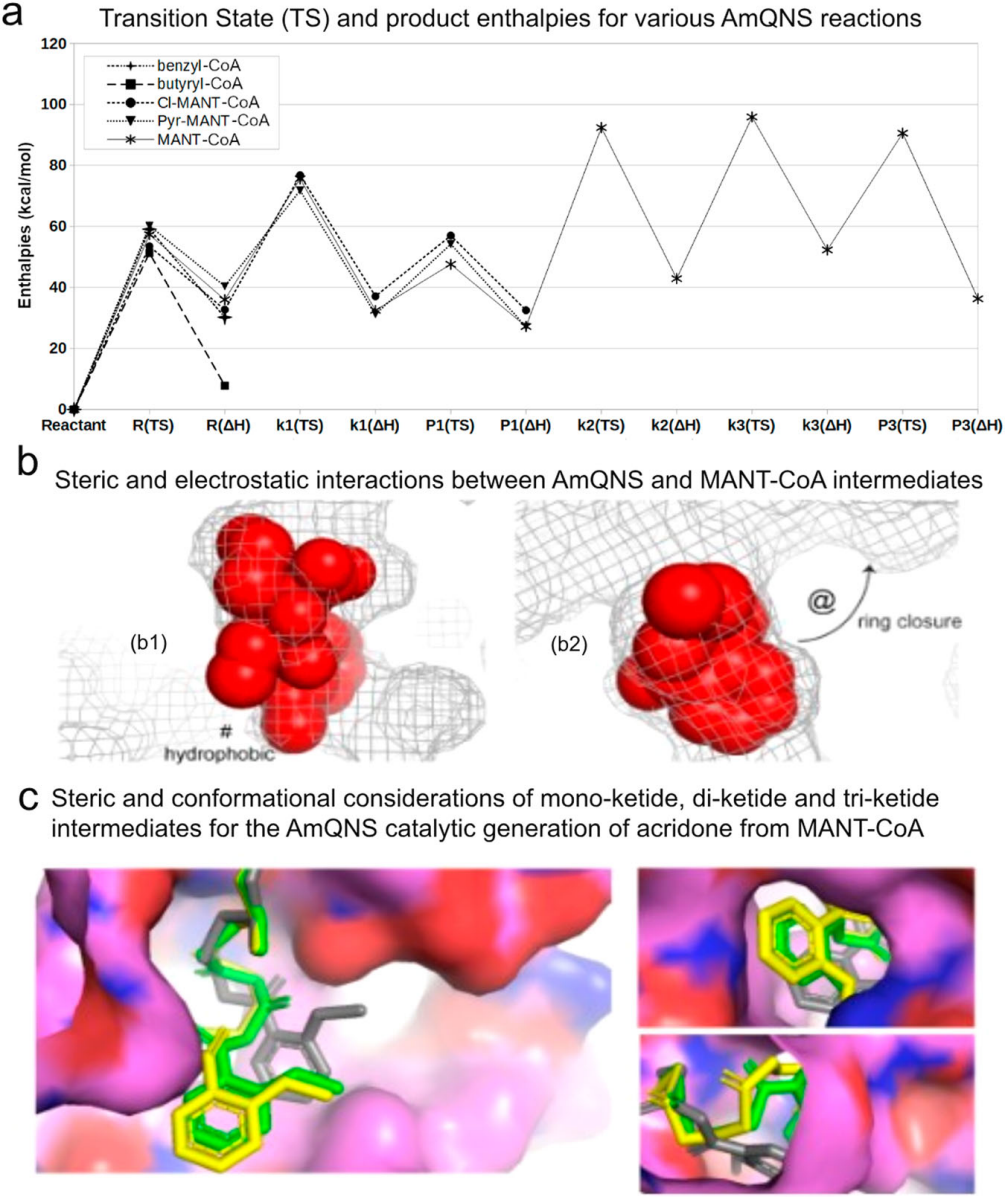
AmQNS mediated synthetic diversity based on the structure conformations. **a** Relative transition state (TS) and product enthalpies for various AmQNS reactions, relating to five distinct core substrates named in the legend (upper left; Cl-MANT refers to a chloromethyl analog to MANT; Pyr-MANT refers to a pyridinyl analog to MANT, with the heteroatomic N located para to the methylamine). Specific reaction state enthalpies are quantified for initial binding (R), specific polyketide insertions (k1, k2, k3) and product cleavages after 1st (P1) and third (P3) ketide insertions. **b** Steric and electrostatic interactions between the AmQNS receptor (mesh) and MANT-CoA-based intermediates (spheres) for the ketide insertion (shown in left) and monoketide product forming (shown in the right) transition states. Steric effects relating to the bulky chlorine atom are represented as ‘@.’ **‘**#’ indicating the hydrophobic pocket. **c** Steric and conformational considerations of mono-ketide (yellow), di-ketide (pink) and tri-ketide (white) intermediates for the AmQNS catalytic generation of acridone from MANT-CoA substrate, as viewed from a cross-section of the whole receptor, the channel through which malonyl-CoA co-substrate enters, and the product release channel.

Next, we report acridone-specific reaction steps (Fig. 5a) and the second and third ketide insertions are predicted to be somewhat less favorable kinetically and thermodynamically compared to the first ketide insertion shown in Fig. 4. In contrast, the final acridone ring closure is expected to have a higher activation barrier than the quinolone product formation but a more favorable reaction enthalpy. Finally, we investigated whether AmQNS is better suited for quinolone or acridone production, and we propose that the key difference between the two reactions may be a matter of stoichiometric control, with an excess of malonyl-CoA favoring acridone and tight stoichiometry favouring quinolone. Furthermore, reducing steric bulk by altering Leu 263 or Ser 132 could enhance throughput of both products, indicating that specific amino acid changes could be used to impact enzymatic product selectivity. For instance, the previously studied AmQNS mutants MSD1 (double mutant, S132T/A133S) and MSD2 (triple mutant, S132T/A133S/V265F) had drastically narrowed active site cavities when compared to the wild-type AmQNS. MSD1 demonstrated chalcone-forming activity with p-coumaroyl-CoA like the typical chalcone synthase, whereas MSD2 did not^14^. Since none of the mutants prefer MANT-CoA as starter substrate, the two amino acid alterations S132T and A133S influenced the enzyme’s substrate selectivity.

It is inherently challenging to fully characterize how type III polyketide quinolone synthases achieve such impressive synthetic diversity from relatively minor structural variations among different enzyme families. Here, we primarily focus on enzymatic steric and electrostatic selectivity for binding other core substrate units (e.g., relative favorability for specialized binding units such as coumaroyl-CoA, benzyl-CoA, acetyl-CoA, MANT-CoA, versus a universal capacity to malonyl-CoA as a substrate or co-substrate), and the amount of space available to accommodate larger numbers of incrementally inserted ketide units. We determined quantum chemical transition states to compare the relative kinetic barriers^41^ and thermodynamic enthalpies^42^ for the initial complexation of MANT-CoA, benzyl-CoA, butyryl-CoA and coumaroyl-CoA. Similar characterization was done, in the case of MANT-based reactions, for the first ketide insertion, the single-ketide quinolone product formation, the second and third ketide insertions, and the triple-ketide acridone product.

The initial complexation barrier showed little variation among primary substrates, implying that steric dependencies play a minor impact at this stage (Fig. 5a). However, steric and electrostatics do appear to discriminate somewhat in the stability of the resulting bound intermediates. Specifically, the pyridinyl analog to MANT-CoA has less stabilization than the others because it places the slightly polar aryl nitrogen directly within a hydrophobic pocket delimited by Ile 254 and Pro 375 (‘#’ in Fig. 5b). Simultaneously, the sole flexible substrate (butyryl-CoA) can conformationally adapt to this pocket in a stabilizing manner. Proceeding from the mono-ketide intermediate (k1) to the monoketide product (P1) reflects minimal difference among the three analogs of MANT-CoA, with the exception that the chloromethyl compound has a higher barrier to ring closure, due to steric effects relating to the bulky chlorine atom.

Next, in Fig. 5a, it showed the quantitative reaction profile of secondary and tertiary ketide insertions by which the monoketide intermediate may progress toward the acridone product (P3). It is worth noting that these latter ketide insertions are predicted to have higher barriers (>50 kcal/mol) than the first insertion (~40 kcal/mol). From a computational perspective, this trend is rationalized by higher conformational strain evident in the di-ketide and tri-ketide units relative to the mono-ketide (Fig. 4b), as opposed to issues relating to the approach of malonyl-CoA co-substrate (for which there is ample space, as shown in Fig. 5c) or the situation of the MANT group (Fig. 4b). Based on this, we propose that AmQNS structurally favors the smaller quinolone product’s production and might thus only produce acridone under conditions of higher malonyl-CoA concentration.

### In-silico mutagenesis suggests potential implications on synthetic selectivity

**Fig. 6:**
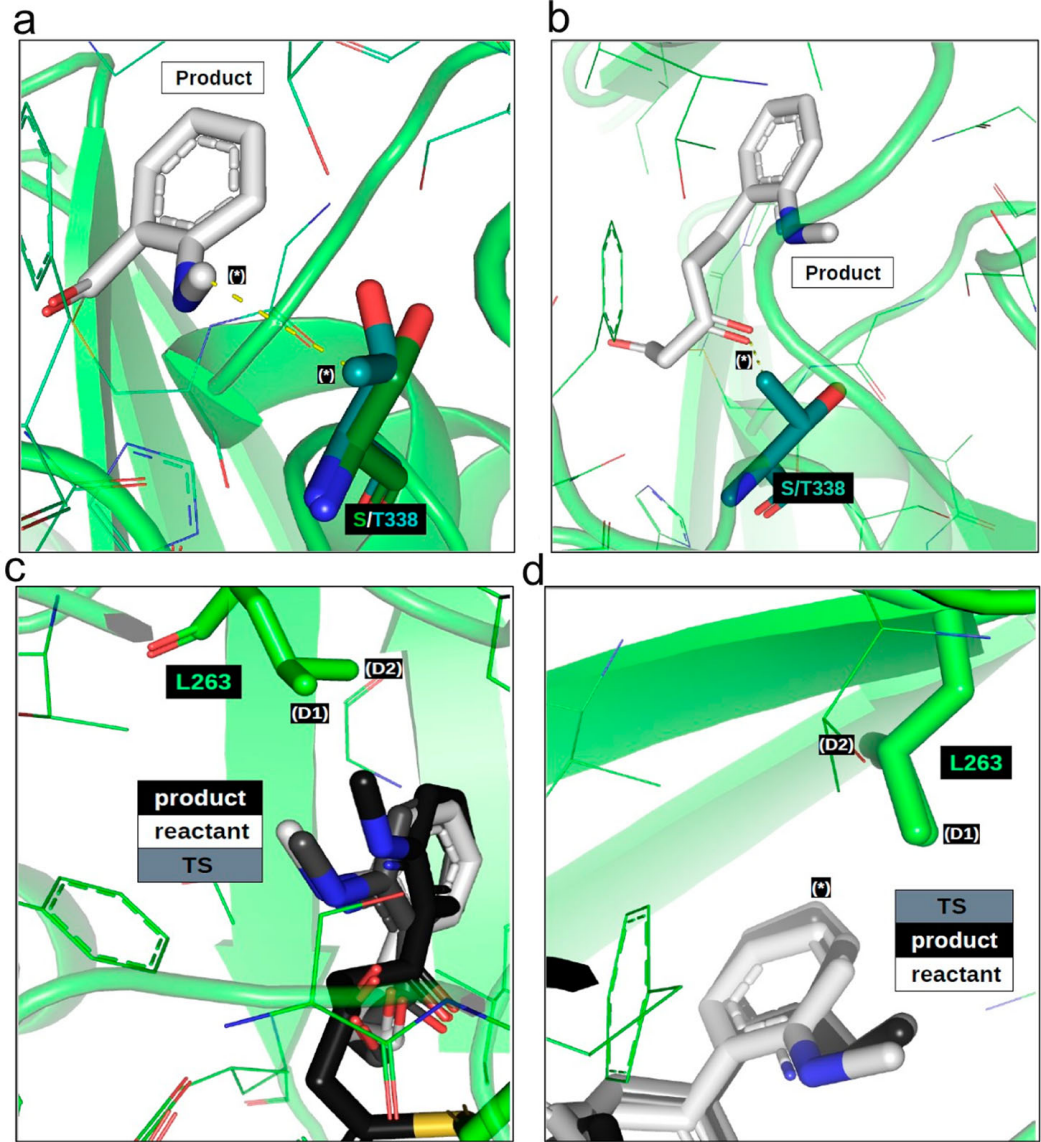
Potential implications of S338 -> T338 and L263 -> I263 mutations. **a** The initial MANT-binding product is sterically closer to position 338 than corresponding reactant or transition state structures. Threonine has a favorable methyl/methyl nonpolar contact (*) that may stabilize the MANT bound product, however steric constraints may negate this S338 → T338 benefit for bulkier substates. **b** While the S338 → T338 mutation may benefit initial MANT complexation and may be neutral for subsequent quinolone production, the methyl group of a threonine is predicted to clash (*) with polyketide intermediates required for acridone or larger polycyclics. **c** Acridone synthesis requires 2nd and 3rd ketide insertion reactions. During the 2nd insertion, a shifting degree of steric contact occurs between the substrate and the two delta methyls of L263 (D1) and (D2)). The ketide insertion product is predicted to have favorable contact with (D1) by the MANT methyl, but minimal contact with (D2). Reactive precursor structures approaching the transition state, however, are predicted to have a minor clash between (D2) and the MANT aromatic, which might slow the reaction and increase the activation barrier. These kinetic impediments may be lessened by a L263 → I263 mutation. **d** Minor contact variations occur between the MANT group and the two delta methyls of L263 (D1) and D2) during the formation of ketide insertion products. Steric constraints in the 3rd insertion are predicted to reduce the magnitudes of these contact variations compared to the 2nd insertion, but these variations may still have a relevant effect on 3rd insertion reactive kinetics. Although both the transition state and product structures exhibit favorable interactions between the MANT methyl and (D1), a slight steric clash during the transition state pushes the substrate aromatic into a more strained position relative to either the reactant or product conformation (*). This theoretical clash might be alleviated by a L263 → I263 mutation.

To further understand how mutations of functionally important amino acid residues alter the synthetic selectivity of AmQNS, in silico mutation studies were done. We suggest that structural modifications to the AmQNS enzyme [e.g., potentially mutating Leu 263 into a smaller valine or alanine (L263 -> V263 or L263 -> A263 mutation) or removing the methylamine clash by mutating Ser 132 into a glycine (S132 -> G132)] might favor both the quinolone and acridone product formation, potentially speeding the production of either while not necessarily affecting the relative ratios of quinolone and acridone product. In the initial mutation experiments, we speculated that L263 -> V263 or L263 -> A263 mutation might favor the quinolone or acridone production but more careful delving into the analysis consistently suggested that isoleucine (I) may be a more fundamentally selective alternative, and that valine (V) might non-selectively diminish reactivity for both quinolones and acridones. Mutation screening also did not suggest many promising mutations, however S338 → T338 could boost quinolone production at the expense of larger initial substrates (Fig. 6). L267 → V267 might improve quinolone and acridone kinetics but might potentially reduce enzyme specificity. N336 is electrostatically useful for steering the transition state. While checking, even though N336 → S336 mutation might provide better TS stabilizing H-bonds for MANT-conversion, such a mutation might reduce enzyme specificity. L263 → I263 suggest boosting the acridone production by stabilizing the transition state (Fig. 6). Analysis also suggests S338 -> T338 may enhance quinolone production while decreasing acridone conversion and L263 -> V263 may enhance acridone production relative to quinolone. More details of in silico mutations and their implications are given in Supplementary Fig. 11.

In the future, we will compare analyses for non-MANT (e.g. Feruloyl-CoA, Hexanoyl-CoA, etc.) substrates. Furthermore, the specific structures available for each of the five (for quinolone) transition states or five (for acridone) reaction steps may be rigorously evaluated to determine which ligand-receptor amino acid contacts are favorable or unfavorable. Also, determine what sorts of mutations could be proposed to substantially alter favourability in a manner that could influence existing AmQNS enzymatic activity and potentially engineer product specificity. These all studies will contribute to prospective future investigations in directed evolution-based studies that replicate Darwinian selection in the laboratory^43,44^ and further metabolic engineering, with the goal of producing specialized metabolites. Directed evolution has already demonstrated its efficacy in enhancing the stability and functionality of type III PKS enzymes. For example, Zha et al.^45^ successfully improved the productivity of PhlD (a type III PKS from *Pseudomonas fluorescens*), through the application of directed evolution. Therefore, these findings have the potential to provide valuable insights and enhance enzyme productivity through the implementation of directed evolution in future engineering endeavors.

## Materials and methods

### Homolog identification and phylogenetic analysis

To identify the potential homologs for AmQNS (UniProt ID: M1HE54), complete predicted proteome sequences were retrieved from JGI and (NCBI GenBank). Homologs were also retrieved from The Marine Microbial Eukaryote Transcriptome Sequencing Project database (MMETSP)^46^. The *Arabidopsis thaliana* chalcone synthase (NCBI accession: AT5G13930) amino acid sequence was also used as a query to search of all potential homologs using the Hidden Markov model (HMM)-based tool jackhammer^47^. Evolutionary genealogy of genes: Non-supervised Orthologous Groups (eggNOG) mapper was used for hierarchical resolution of orthology assignments^48^. Finally, the SMART and Pfam databases were employed to identify conserved domains present in type III PKS from different organisms^49,50^, i.e. both SMART and Pfam databases were merged, and redundant domains were filtered-out and used Hidden Markov model (HMM)-based tool hmmscan (https://github.com/EddyRivasLab/hmmer). Only sequences with the catalytic or conserved domain of the references were retained for further analysis. The identified homologs and the respective details are given in Supplementary Data 1–4. All identified homologs were aligned using MAFFT^51^ and ambiguously aligned regions were excluded for further analysis using trimAl software^52^. Alignments were tested using ProtTest v3^53^ to choose an appropriate model for nucleotide substitution. Two separated Maximum likelihood (ML) phylogenetic trees were computed using RAxML-NG^54^ and IQ-TREE2^55^. ML-based phylogenetic analyses were performed using 1000 bootstrap replicates. The supporting values from both software were noted on the ML tree.

### Large-scale AmQNS expression and purification

The QNS gene from ‘*A. marmelos*’ (AmQNS) was cloned into pET32b, as explained by Resmi et al.^14^. To successfully express the fusion protein, transformed *Escherichia coli* BL21(DE3) cells were first verified by colony PCR (Supplementary Fig. 10a, b), and the confirmed colonies were cultured at 37 °C in Luria-Bertani (LB) medium with ampicillin (100 mg/ml) until they reach the exponential phase (OD600 - 0.6). Isopropyl 1-thio-D-galactopyranoside (IPTG, 0.4 mM) was employed to induce AmQNS expression, and the cells were further incubated at 28 °C for 5–6 h. The cells were harvested by centrifugation (5000 *g*, 30 min, 4 °C), and the pellet was resuspended in KPO_4_ buffer (50 mM, pH 8) containing NaCl (0.1 M), imidazole (40 mM), and lysozyme (750 μg/ml). The lysate was sonicated (amplitude: 35%, 3 s on, 5 s off, 30 min) on ice after being incubated for half an hour on ice. The lysate was then centrifuged (10,000 *g*, 30 min) and the supernatant was then loaded to a Ni-NTA (nickel-nitrilotriacetic acid) affinity column equilibrated with KPO_4_ buffer (50 mM, pH 7.9) containing NaCl (0.5 M) and imidazole (40 mM). All phases of protein purification were carried out at 4 °C in cold room. In the resuspended condition, the system was allowed to bind at 4 °C (1–2 h). The recombinant protein was eluted in 15 mM KPO_4_ (pH 7.5) buffer containing 500 mM NaCl, 500 mM imidazole, and 10% glycerol after a lengthy wash of the column with the same equilibration buffer (10 column volumes). Purified recombinant AmQNS (61.28 kDa) fractions (fusion protein containing an N-terminal Trx-S-His fusion tag) were concentrated (Amicon-Ultra centrifugal filters, 10 kDa cut-off) and overnight enterokinase cleavage was performed to remove the fusion tag (Supplementary Fig. 10c). Size exclusion chromatography on a Superdex 200HR (10/100 GL) column (GE Healthcare) in HEPES-NaOH buffer (20 mM, pH 7.5) containing NaCl (100 mM) and dithiothreitol was used to further purify the AmQNS protein solution to homogeneity (DTT, 2 mM). The purified AmQNS (42.8 kDa) fractions were further concentrated (10 mg/ml and 20 mg/ml stocks) by using the same HEPES buffer. SDS-PAGE was used for qualitative analysis (Supplementary Fig. 10f), and the quantity was also calculated using the NanoDropTM1000 spectrophotometer (Thermo Scientific, Wilmington, DE) at an optical density (OD) ratio of 260/280 (and default protein absorbance values for 0.1%. i.e., 1 mg/mL). MALDI-TOF MS analysis was used to determine the protein’s homogeneity and mass accuracy (Supplementary Fig. 10d, e).

### AmQNS crystallization by microbatch method

For crystallization trials, we used both hanging drop and microbatch methods with varying protein concentrations (range of 5–20 mg/ml) and drop ratios (protein-precipitant ratios of 1:1, 2:1, 1:2, 3:1, 1:3), however, only the microbatch approach (using 10 mg/ml protein concentration, 1:1 drop ratio) resulted in nucleation and crystal formation. In addition, we were unsuccessful in acquiring any crystals when using frozen protein. Therefore, we exclusively used freshly purified proteins for the purpose of crystallization. Further these successful conditions were optimized (using additives) to get diffraction quality crystals. Co-crystallization trials were also performed in the presence of its natural CoA substrates ‘*N*-methyl anthraniloyl CoA (MANT-CoA)’ and byproduct CoASH. A solution containing the substrate/byproduct was directly added to the concentrated protein solution to a final concentration of ~2 mM and incubated in ice for an hour prior to crystallization trials by micro-batch method. The diffraction quality of these substrate-bound crystals was also optimized by adding additives, varying drop sizes and protein and/or precipitant concentrations. The crystals appeared within a span of 2-3 weeks with approximate dimensions of 0.1 mm×0.1 mm×0.1 mm (Supplementary Fig. 10g). Since the MANT-CoA was not clearly resolved in crystals from co-crystallization experiments, we proceeded to carry out soaking experiments. Here, the AmQNS native crystal was soaked (30 min) in ligand solution, which was prepared in the same crystallization condition (including 0.1 M cadmium chloride hydrate as additive) supplemented with 2mM *N*-methylanthraniloyl-CoA. AmQNS crystals were obtained in three different forms (native, substrate and byproduct bound) when using 1:1 drop ratio (using 2 µL of protein and 2 µL of precipitant (0.1 M HEPES 7.5, 1.4 M Sodium citrate tribasic + additives)). Following the addition of a suitable cryoprotectant (20% glycerol), crystals were picked with a nylon loop, flash frozen in liquid nitrogen and data were collected. Successful crystallization and soaking conditions from the experiments are given in Supplementary Table 1.

### X-ray collection, processing, and refinement

After obtaining diffraction-quality crystals, the crystals were cryoprotected (20% glycerol crystallization reservoir solution) by plunging them into liquid nitrogen using a fine-gauge wire micro loop. Data from the native apo and CoASH bound crystals belonging to the space group H32 were collected at the Molecular Biophysics Unit (IISc, Bangalore) using a MAR 345 image-plate detector mounted on a Bruker MICROSTAR ULTRA II Cu Kα rotating anode X-ray generator (wavelength of 1.54179 Å). For collecting the high-resolution data, the detector distance was adjusted to 200 mm. All data were collected cryogenic temperatures (100 K). AmQNS native crystal diffracted up to 1.85 Å and all the data collection statistics are given in Table 1. iMosflm was used to process the diffraction images^56^, and data were scaled, merged and converted into structure-factor amplitudes using SCALA^57^, POINTLESS, AIMLESS and TRUNCATE in the CCP4 suite^58–60^.

The structures were determined by the molecular replacement method to 1.85–2.35 Å resolution in space group H32 with PHASER^61^ using the structure of acridone synthase from *Citrus microcarpa* (PDB: 3WD7; 93% sequence identity) as the search model^17^. The structures were subsequently refined using REFMAC5^62^, along with multiple rounds of manual model building using COOT v0.7.1^63,64^. The addition of the ligands and water atoms was performed by PRODRG^65^ or eLBOW^66^. The possibility of alternate ligand conformations was also evaluated before finalizing the ligand fitting. CheckMyMetal^31^ (CMM, https://cmm.minorlab.org/) server was used to validate the metal binding sites present in macromolecular structures. The final structure refinement of the native structures was performed in PHENIX^67^. Images of the protein structures were generated using PyMOL Licenced academic version^68^. The refined models were validated by PROCHECK^69^ and the MOLPROBITY^70^. All structural models were manually built, refined, and rebuilt with REFMAC5 or PHENIX and COOT.

### Structural analysis

The refined protein structures were evaluated using MolProbity with the PHENIX server^67^ and wwPDB server^71^. Initial structural alignments were performed in ALIGN (Pymol^68^) and mTM-align^72^. Further, to visualize the trend of sequence conservation, we employed ClustalW^73^. The patterns of conservation in the sequence and structure were visualized using ESPript and ENDscript 2.0^74^. The neighbor-joining method^75^ was used to construct a structural phylogram. Electron density 2Fo-Fc and Fo-Fc maps were converted to CCP4 format using the FFT (Fast Fourier Transform) module of the CCP4 suite v7.0^76^, and the maps were visualized in PyMOL using the command line option (contoured at 1.0 sigma around the selection site within 1.6 Å of the selected atoms). The electrostatic properties of AmQNS were calculated using APBS using the PyMOL plugin. PDB2PQR Version 2.0.0^77^ was used to convert the PDB files into PQR files. To obtain the detailed characteristic features of the surface pockets and interior voids of AmQNS, CASTp (Computed Atlas of the Surface Topography of Proteins) was used^78^. The default probe radius was used (1.4 Å) and the protein secondary structure and protein‐ligand interactions (determined using LIGPLOT) were analyzed using (PDBsum)^79^. Polder maps were created in PHENIX v1.20.1^67^ (i.e. omit selection ‘Chain A and resseq 401’ for specifying MANT-CoA ligand). Potential cysteine modification sites on AmQNS were predicted using the (pCysMod server)^28^. The relative position of functional and structural importance among the protein homolog sequence sites was estimated using Evolutionary Trace (ET; http://evolution.lichtargelab.org/). All figures were prepared using the academic version of PyMOL v2.4.1^68^.

### Surface plasmon resonance (SPR) based AmQNS- substrate interaction studies

ProteOn XPR array system^21^ and ‘GLM’ sensor chip was used for the SPR interaction studies. AmQNS showed strong responsiveness (L3 - 9962 RU, L4 - ~7000) when immobilized on the ‘GLM’ sensor chip (Supplementary Fig. 1). Here, amine coupling works, where the amine groups present in the AmQNS covalently bind to the chemically activated carboxyl groups of the dextran molecules. Channel L3 and L4 were used for immobilization of protein while L2 was used as reference. The extent of non-specific interactions was eliminated or reduced by optimizing the buffer conditions. Furthermore, ligand stability on the GLM biosensor over time was checked over a period of 30 days and was found that it is active, by resulting in quantifiable interactions. This demonstrates that the AmQNS immobilization onto the GLM sensor surface does not limit the functionality, confirming the use of this SPR label-free technology to study its interaction pattern with different acyl-CoA substrates. *N*-methylanthraniloyl-CoA was purchased from TransMIT (Plant MetaChem, www.plantmetachem.com), whereas all other substrates were purchased from Sigma-Aldrich (www.sigmaaldrich.com).

Following AmQNS immobilization and its stability screening, all binding studies were done at 30 °C. The SPR-based system measures the changes in refractive index to investigate the direct interaction between AmQNS and different CoA substrates. The analytes (substrates) were injected over the surface of the chip, and any binding between the two resulted in a change in surface mass, which is recorded and measured as a change in refractive index. In our experiments, AmQNS was captured on the surface of the GLM sensor chip and used to screen the preferred substrates (acyl-CoA’s) in the presence and absence of malonyl-CoA. We could not find any interaction in the absence of malonyl-CoA, and it was quite interesting to note that the binding modes of these CoA substrates to AmQNS are influenced by the presence of the malonyl-CoA, which is the extender during the biochemical reaction of polyketide formation. The sensogram was prepared by processing the data (after subtraction of L2 responses (reference channel). Baseline drift due to the bulk refractive index change, non-specific binding, matrix effects and injection noise were also corrected using the reference spots. Further, the responses obtained from the AmQNS-small molecular interactions at different concentrations were fitted using the Langmuir 1:1 biomolecular interaction model using the ProteOn Manager software version 3.1.0.6 (Bio-Rad, USA). Equilibrium dissociation constants (*K*D) were calculated from the ratio of the association and dissociation rates.

### Molecular simulation studies

The AmQNS polyketide synthase active site structural model was constructed from an AmQNS crystallographic model (PDB ID: 6L5U) in PyMOL^80^ as the set of all amino acids with at least one atom residing within 12.0 Å of the catalytic Cysteine (C164). Peripheral peptide chain termini were neutralized by simple protonation to neutral amine and aldehyde structures. Specific ligands (MANT-CoA and malonyl-CoA) were constructed in situ using PyMOL by referring to the EthSH group of the co-crystallized CoASH ligand. For computational efficiency, the bulk of the conserved CoA moiety (i.e., all except for those mentioned above -EthSH moiety) was removed from each ligand. Transition state calculations were performed using MOPAC 2016^81^, via the PM7 parametrization^82,83^. Due to the exceptional complexity of the potential energy surface (PES)^84,85^, it was necessary to manually perform transition states by employing constraints to implement a stepwise approach between reacting atoms.

We also calculated transition states for MANT-CoA binding to AmQNS. An initial step size of 0.4 Å was used for the initial (distant) ligand approach until the approaching atoms were within 1.0 Å of the expected covalent distance. A step size of 0.1 Å was employed to capture subtle structural and energetic effects. All receptor backbone atoms were held rigid to prevent spurious peripheral conformational shifts from quantitatively overwhelming covalent energetics, as were all side chains except those participating directly in enzyme reaction function. In addition, we also determined quantum chemical transition states to compare the relative kinetic barriers and thermodynamic enthalpies for the initial complexation of MANT-CoA, benzyl-CoA, butyryl-CoA and *p*-coumaroyl-CoA.

### Structure-based contact-dependent mutagenesis studies

PyMOL^68^ was used to map residue-specific substrate-enzyme contact surfaces in the quantum chemical reaction coordinate profiles reported in this study for each of the nine reactions that collectively comprise the AmQNS synthesis steps for quinolone and acridone production. These contact surfaces were sensitive to the solved positions of all protons and heavy atoms, and used a 1.5 Å probe to detect all substrate-residue spatial proximities less than 3.0 Å. From the above contact detection protocol, contact traces were assessed for specific enzyme residues with appreciable substrate contact, making the qualitative assignment of ‘minor contact’ (pink) for specific instances with non-zero residue/substrate contact surfaces with areas of less than 0.5 Å2, and major contact (red) for contacts with areas greater than or equal to 0.5 Å2.

### Statistics and reproducibility

X-ray crystallization experiments were performed multiple times (more than three), and data collection statistics are included in Table 1. SPR assays were performed in two or three independent trials. The procedures and supplementary files contain comprehensive information regarding the experimental particulars and the data used.

### Reporting summary

Further information on research design is available in the Nature Portfolio Reporting Summary linked to this article.

### Supplementary information

Supplementary Information

Description of Additional Supplementary Files

Supplementary Data 1-4

Supplementary Movie 1

Supplementary Movie 2

Supplementary Movie 3

Reporting Summary

## Data Availability

Coordinates and structure factors for the above-mentioned AmQNS structures have been deposited in the Protein Data Bank (accession codes: 6L5U, 6L7J, and 7CCT).
