# Supplementary material for: Structural and mechanistic insights into Quinolone Synthase to address its functional promiscuity
Source: Commun Biol. 2024 May 14;7:566. doi: 10.1038/s42003-024-06152-2 (PMC11093982; doi:10.1038/s42003-024-06152-2)
Supplement: Supplementary file 1 — Supplementary Information [file 42003_2024_6152_MOESM1_ESM.pdf]

Supplementary Information

(Contains all the supplementary figures and tables associated with the manuscript '*Structural and mechanistic insights into Quinolone Synthase to address its functional promiscuity*')

INDEX

Supplementary Figures: pages 1-16

No	Short Title	Page No
Supplementary Figure 1	AmQNS - *N*-Methylanthraniloyl-CoA binding studies	1-2
Supplementary Figure 2	Structure summary of AmQNS - PDBsum output	3
Supplementary Figure 3	Cartoon representation of active site architecture	4
Supplementary Figure 4	Map of active site and substrate binding residues in the superimposed models	4
Supplementary Figure 5	Comparison of cavity parameters of AmQNS with its nearest homologues	5
Supplementary Figure 6	Cysteine modifications in the structures of native apo AmQNS and in the CoASH bound form	6
Supplementary Figure 7	LIGPLOT representation of AmQNS - N-Methylanthraniloyl-CoA	7
Supplementary Figure 8	CoASH bound AmQNS and LIGPLOT representation	8
Supplementary Figure 9	Chemical structure of MANT-CoA, quinolone/ acridones	9
Supplementary Figure 10	AmQNS fusion protein-expression-purification-crystallization details	10
Supplementary Figure 11	Details of *in silico* mutations (functionally relevant residues) and their implications	11-16

Supplementary Tables: pages 17-20

No	Title	Page No
Supplementary Table 1	Crystallization and soaking conditions for native apo, CoASH bound, and MANT-CoA substrate bound AmQNS.	17
Supplementary Table 2	Potential cysteine modification sites on AmQNS	18-19
Supplementary Table 3	MANT quinolone and acridone kinetics	20

Supplementary Figures

Supplementary Figure 1. AmQNS - *N*-Methylanthraniloyl-CoA binding studies.
AmQNS was successfully entrapped on the surface of a medium capacity 'GLM chip', and its interaction with its most favorable natural substrate, *N*-methylanthraniloyl-CoA (MANT-CoA), appears to fit well into the Langmuir model.

In the Supplementary figure **1 (a-h): -**
a) L2 - reference channel. **b, c**) Channel L3 and L4 were used for AmQNS (50 µg/ml) immobilization (duplicate). SPR responses on the sensor chip surface (L3 - 9962 RU; L4 - ~7000 respectively, i.e., AmQNS was captured on the chip to the maximum final density of 9962 RU). **d, e** and **f**) analyte interaction (*N*-Methylanthraniloyl-CoA) to the immobilized AmQNS at various concentrations and its kinetics. Not all concentrations are shown.

a **b** **c**

d **e** **f**

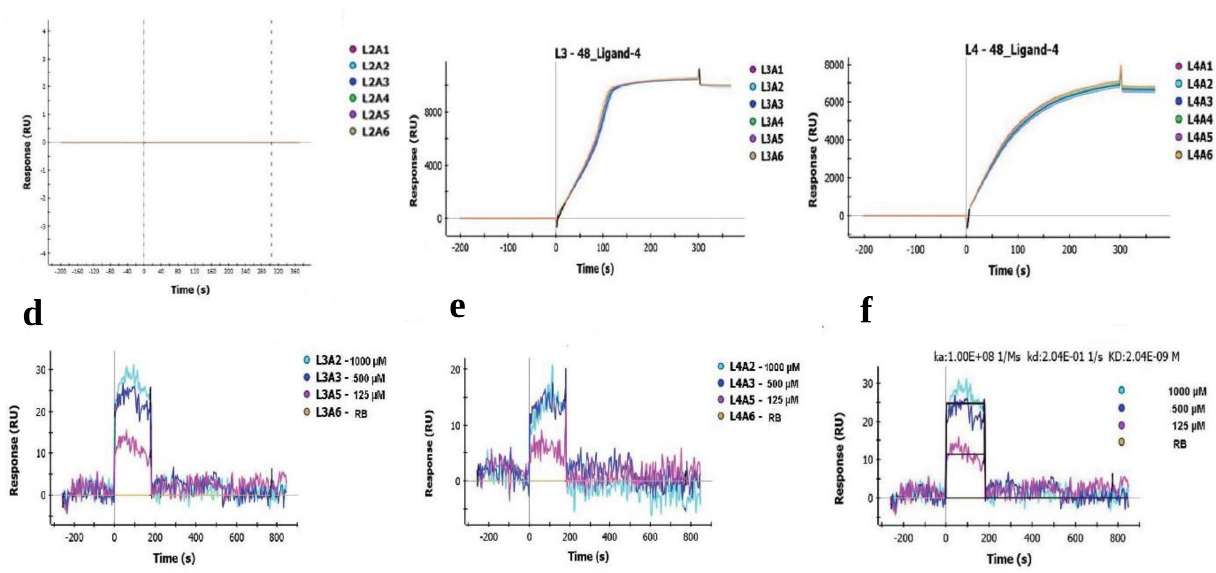

g. Kinetic constraints for AmQNS- different CoA interactions

No	Substrate	K_a(1/Ms)	K_d (1/s)	KD (M) (KD= kd /ka)
1	*N*-Methylanthraniloyl-CoA (MANT-CoA)	1.00E+08	2.04E-01	2.04E-09
2	Feruloyl-CoA	1.00E+08	9.83E-01	9.83E-09
3	Hexanoyl-CoA	9.63E+05	7.03E-03	7.30E-09
4	Palmitoyl-CoA	4.58E+04	4.17E-03	9.11E-08
5	*p*-coumaroyl CoA	5.91E+05	5.95E-02	1.01E-07
6	Sinapoyl-CoA	1.41E+05	1.63E-02	1.16E-07
7	Stearoyl-CoA	1.17E+04	1.93E-03	1.64E-07
8	Malonyl-CoA	7.82E+04	1.34E-02	1.72E-07
9	Myristoyl-CoA	1.30E+04	4.04E-03	3.10E-07
10	Butyryl-CoA	3.61E+04	4.95E-02	1.37E-06
11	Benzoyl-CoA	9.51E+03	2.67E-02	2.80E-06

h. AmQNS -substrate interaction kinetic studies using Surface Plasmon Resonance (SPR). The y-axis shows the KD values in real-time corresponding to the interaction of AmQNS with different substrate-CoAs used. A low KD value correlates to high affinity of AmQNS with the substrate.

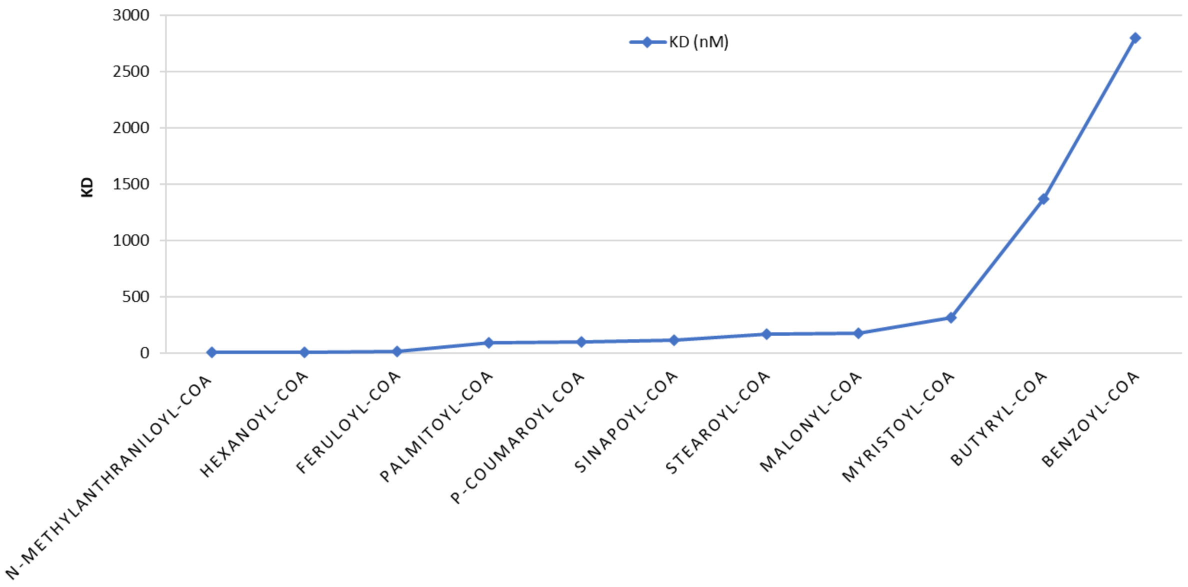

Supplementary Figure 2. Structure summary of AmQNS. Modified PDBsum output showing the secondary structure wiring diagram (computation based on PROMOTIF v.3.0 in pdbsum; accessed ‘pdbsum’ on 29/12/2023 at https://www.ebi.ac.uk/thornton-srv/databases/pdbsum/)

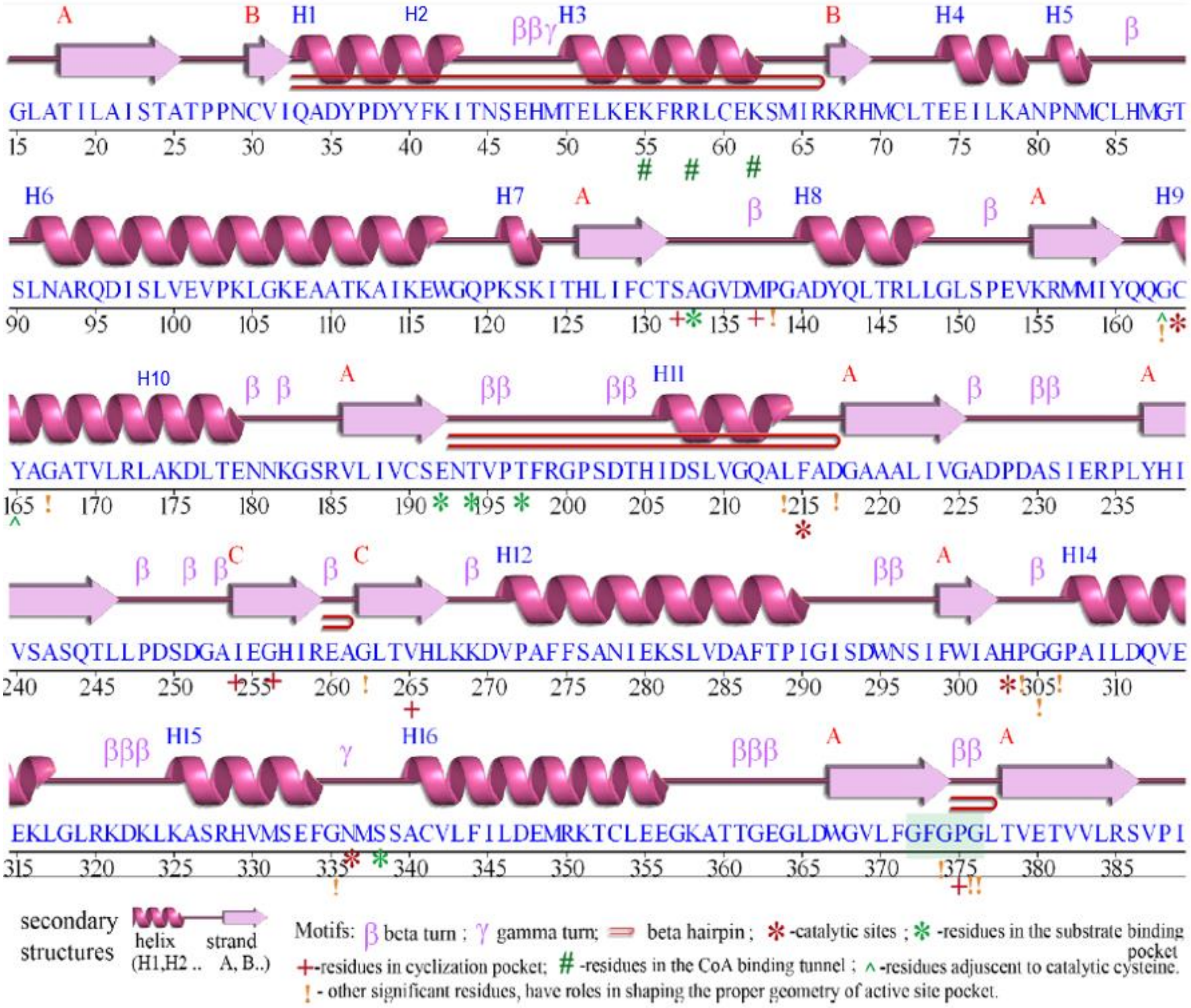

Supplementary Figure 3. Cartoon representation of active site architecture of AmQNS and its nearest homologues.

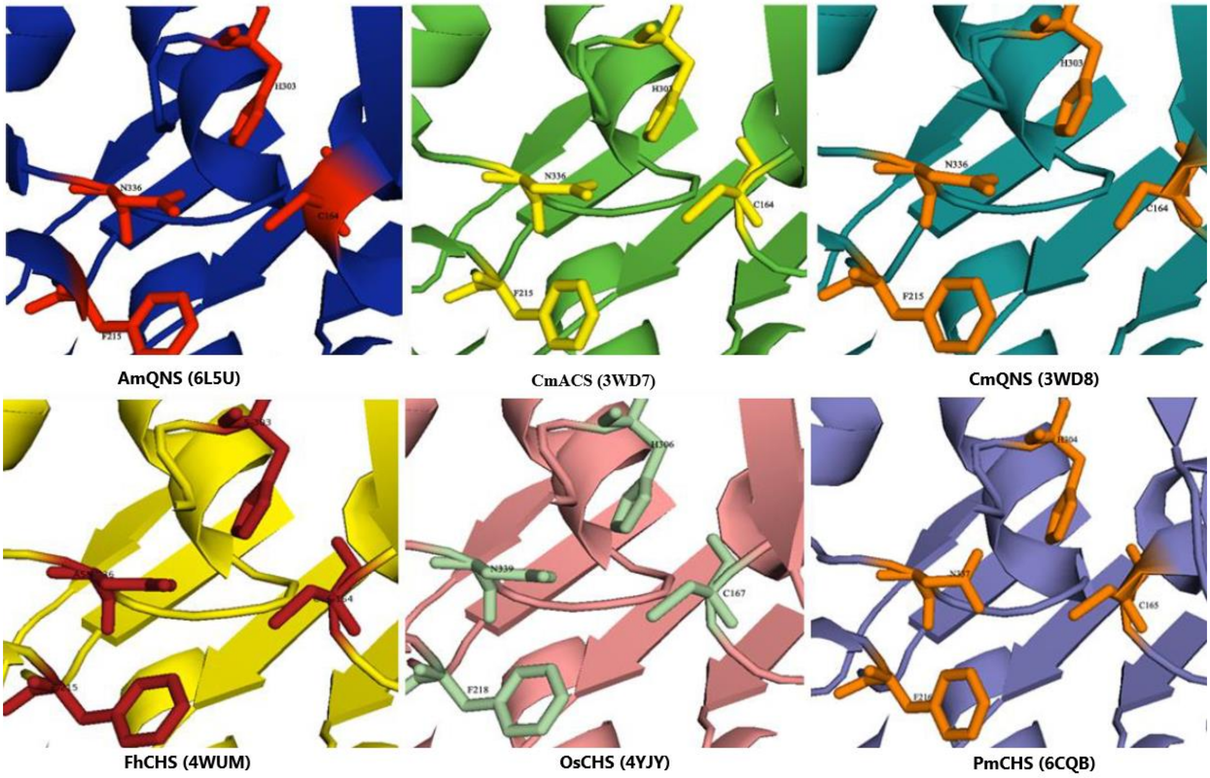

Supplementary Figure 4. 2Fo-Fc electron density map of the active site residues and substrate binding residues in the superimposed models (contoured at 1.0 sigma level).

a) AmQNS superimposed with CmACS. Active site residues of AmQNS represented in orange color, and of CmACS in pink color **b)** Substrate binding residues in the superimposed model. **c)** Superimposition of AmQNS with CmACS and CmQNS.

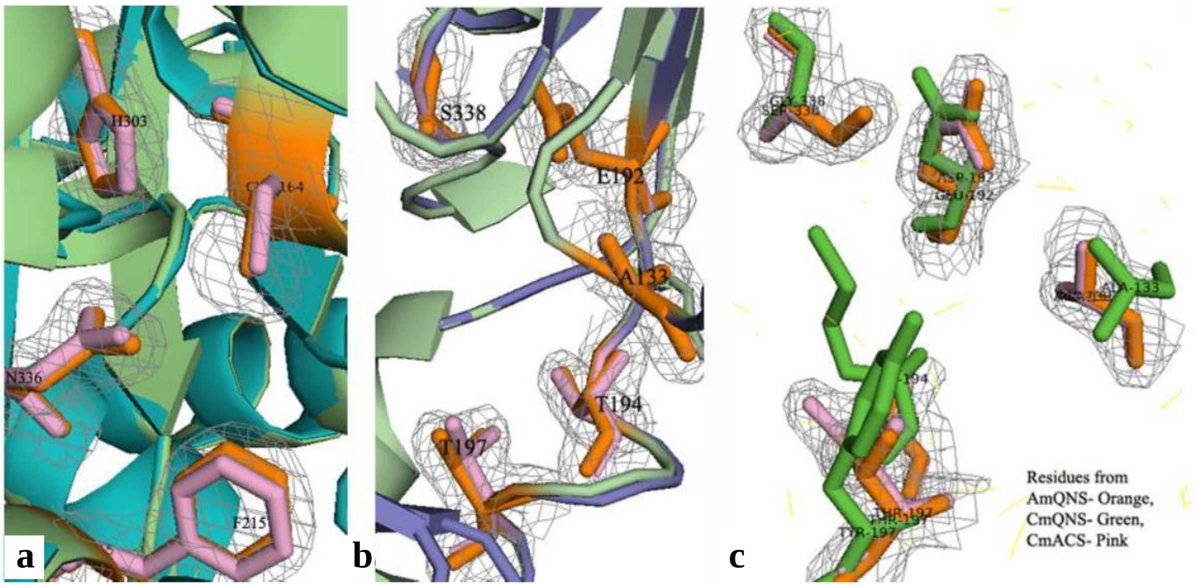

Supplementary Figure 5.

a) Comparison of cavity parameters of AmQNS with its nearest homologues [Abbreviations: AmQNS- Quinolone synthase from *Aegle marmelos*; CmACS – Acridone synthase (ACS) from Citrus x macrocarpa; CmQNS - Quinolone synthase (QNS) from Citrus x macrocarpa; PmCHS – Chalcone synthase (CHS) from *Piper methysticum*; OsCHS - CHS from *Oryza sativa*; MdCHS – CHS from *Malus domestica*: FhCHS – CHS from *Freesia hybrida*; AtCHS- CHS from *Arabidopsis thaliana*].

b) Cartoon representation of AmQNS (6L5U), CmACS (3WD7) and CmQNS (3WD8).

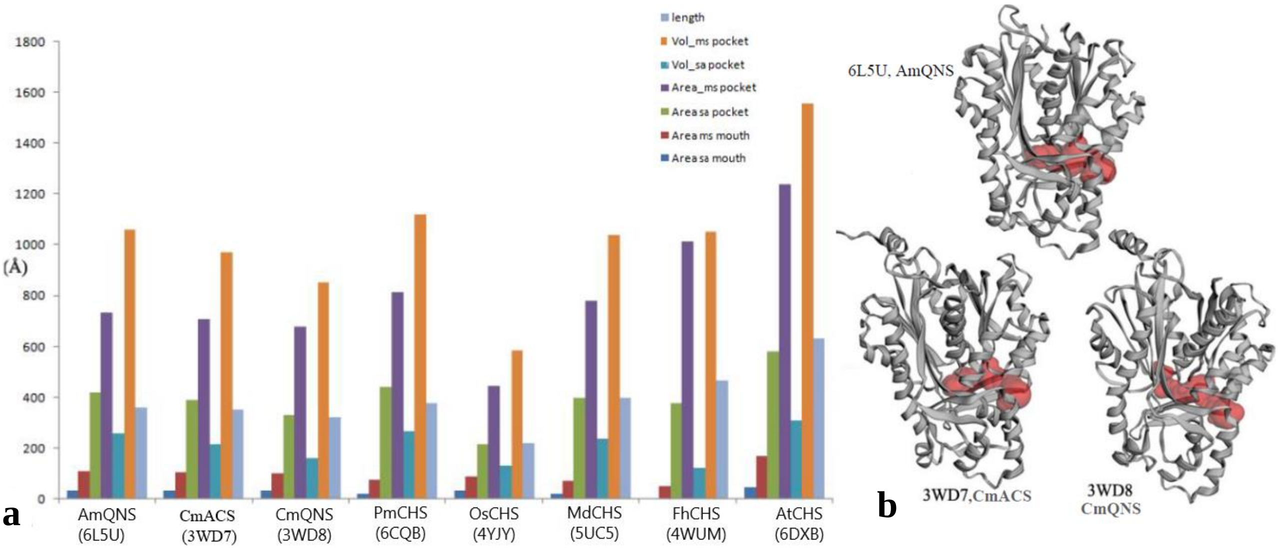

Supplementary Figure 6. Cysteine modifications in the structures of native apo AmQNS and in the CoASH bound form.

a) The catalytic triad of AmQNS native apo structure (PDB ID: 6L5U).

b) The catalytic triad of the CoASH-bound AmQNS structure (PDB ID: 6L7J). The catalytic triads are shown in orange sticks in both **a** & **b**. C164 is modelled with an S-sulfinylation modification.

c) The C71 modelled with S-sulfenylation modification in CoASH bound AmQNS structure. All electron density maps ($2F_o$-F_c, 1.0 σ, blue) and difference map densities (F_o-F_c, 3.0 σ, green) are calculated from structures before modelling the modifications.

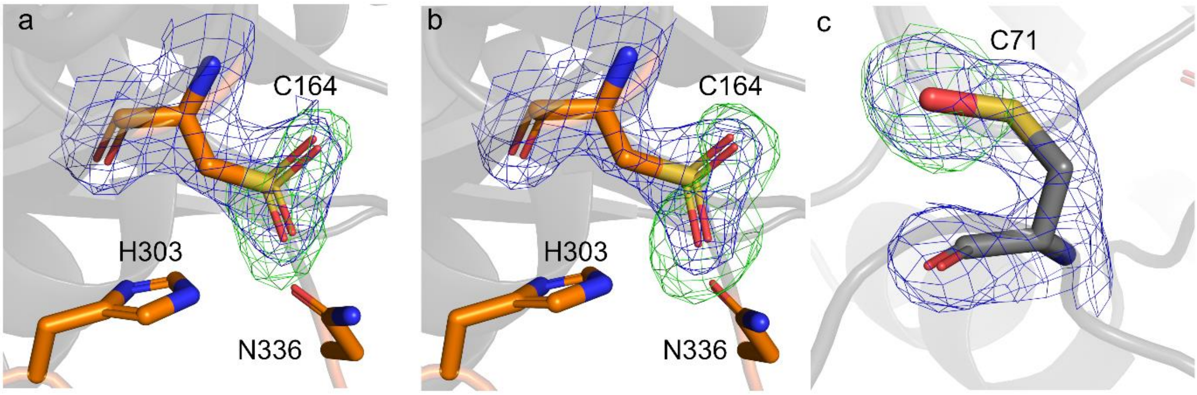

Supplementary Figure 7. LIGPLOT representation of AmQNS - N-Methylanthraniloyl-CoA interactions. Hydrogen bonds between the protein and ligands are highlighted in green dashed lines. (for PDB: 7CCT, Ligand interactions from pdbsum: https://www.ebi.ac.uk/thornton-srv/databases/pdbsum/, dated 26/03/2024)

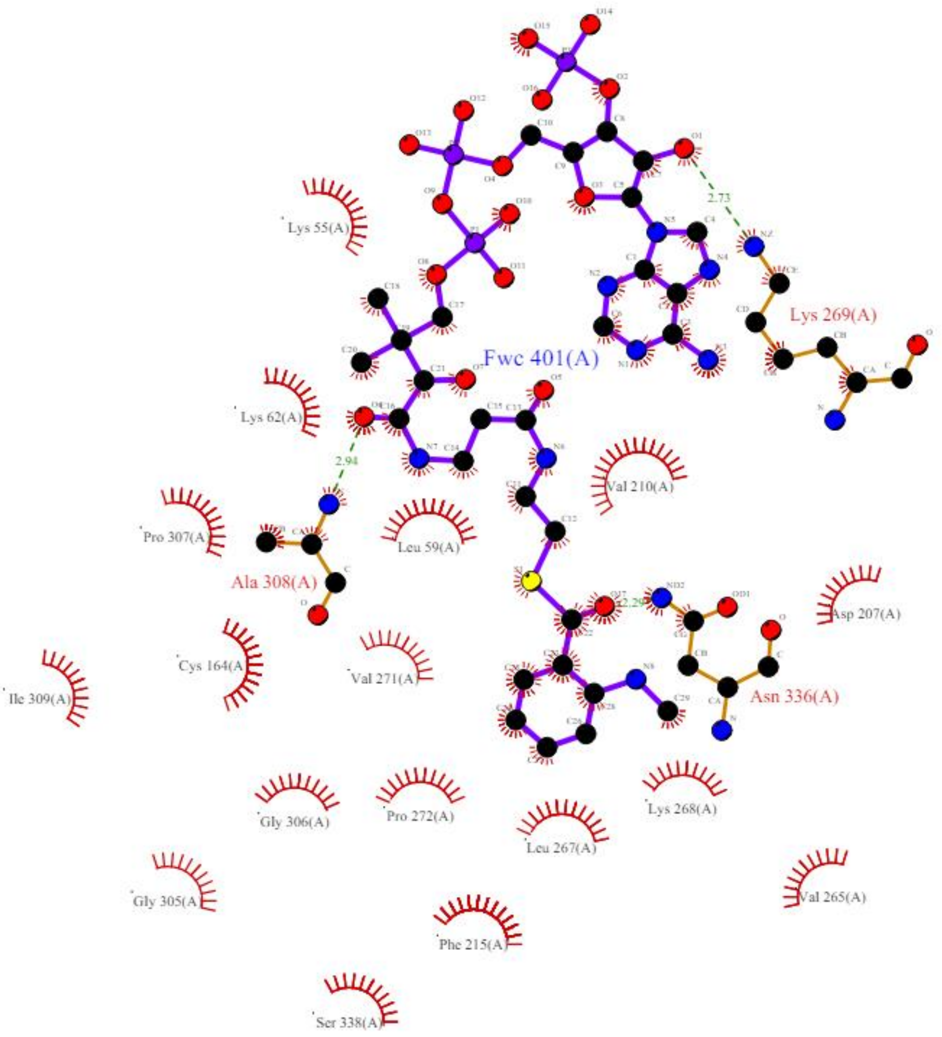

Supplementary Figure 8:

a) CoASH (by-product)-bound AmQNS. Orientation of the ligand molecule in the cavity is depicted. Significant residues highlighted (catalytic sites (C-F-H-N) in orange; substrate binding sites in pink, cyclization residues in yellow, CoA binding residues in pale cyan, Residues shaping the geometry of active sites in blue.

b) Enlarged view of substrate binding pocket above and the position and orientation of significant residues, upon ligand binding is below.

c) LIGPLOT representation of AmQNS - CoASH interactions. Hydrogen bonds between protein and ligands are highlighted in green dashed lines. (for PDB: 6L7J, Ligand interactions from pdbsum: https://www.ebi.ac.uk/thornton-srv/databases/pdbsum/)

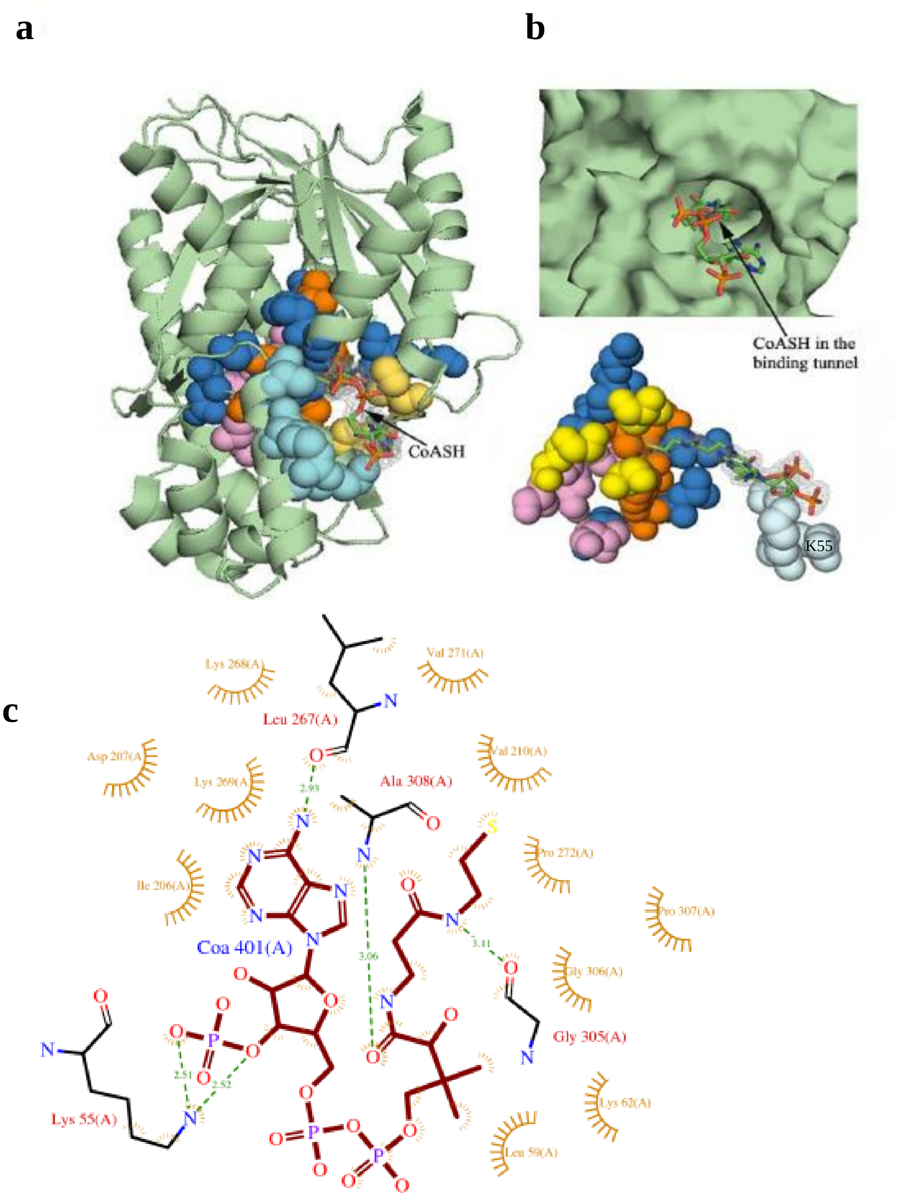

6l7j: Ligplot of interactions with COA

Supplementary Figure 9: Chemical structure of MANT-CoA, quinolone/ acridones

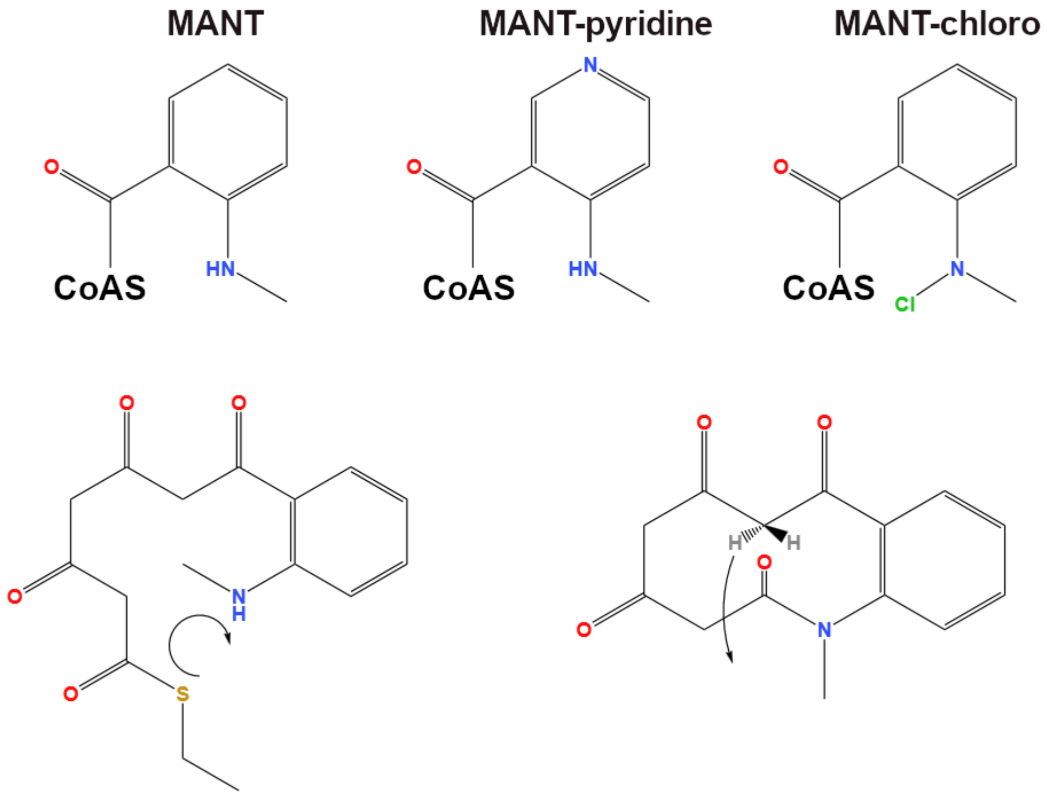

Supplementary Figure 10. a) AmQNS fusion protein sequence (Resmi et al., 2013)[15]**.** Green color represents AmQNS sequences. Details of tags and Enetrokinase cleavage sites are highlighted**.** **b)** Colony PCR of pET32b-AmQNS clones [Lane 1: 1kb ladder, Lane 2-3: AmQNS insert. Lane 4- control]. **c)** PAGE profile showing protein after successful enterokinase cleavage, Lane M- Broad range protein marker, Lane 1- AmQNS fusion protein, Lane 2- AmQNS after Enterokinase cleavage. **d)** The peak in the MALDI chromatogram showing the ~61.28 kDa AmQNS-fusion protein. **e)** MALDI chromatogram of AmQNS after enterokinase cleavage (43.9 kDa). The calculated molecular mass of AmQNS is 42.8 kDa, but with the inclusion of approximately 10 amino acid residues from the vector causing the change in molecular mass up to 43.9 kDa. **f)** SDS–PAGE profile after Size exclusion chromatography [Lane (1-7): purified fractions (17-27 alternate from the sample collector), Lane (8-12): fractions (28-34), Lane 13-marker, Lane 14- Enterokinase cleaved sample before Size exclusion chromatography]. The fractions labelled with arrows were pooled, concentrated using Amicon® Ultra centrifugal filter, and were used for further crystallization experiments. **g**) AmQNS crystals obtained after optimization, with approximate dimensions of 0.1 mm X 0.1 mm X 0.1 mm

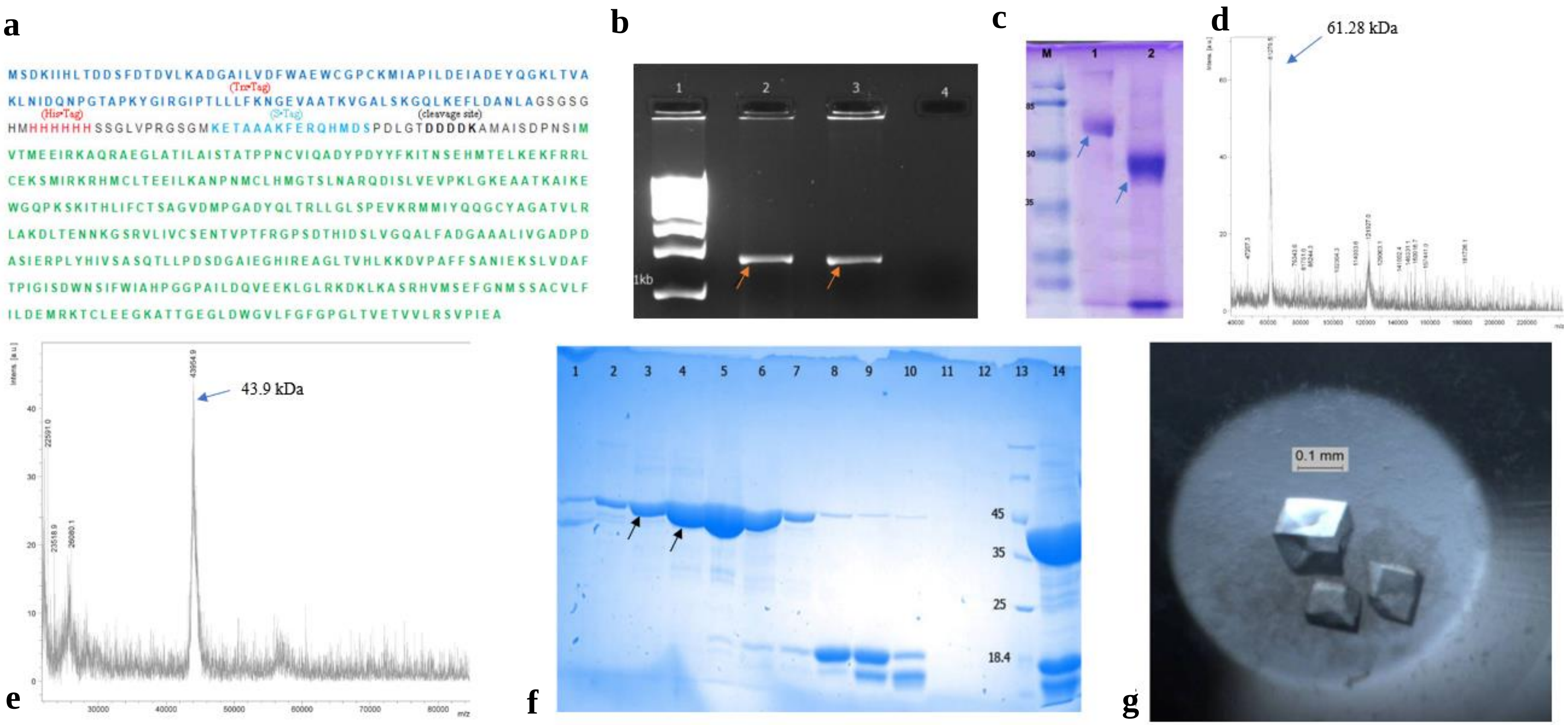

Supplementary Figure 11. Details of in silico mutations (functionally relevant residues) and their implications

EXPLANATION / KEY

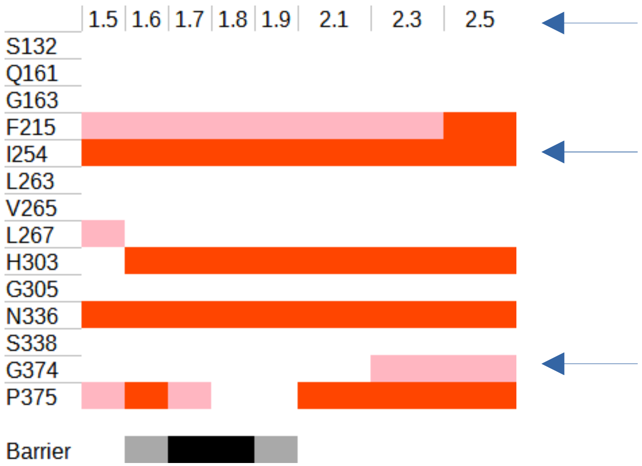

Reaction coordinate (note that dissociative reactions go from left to right; associative go from right to left)

Red implies substantial area close contact surface (3.0 Å) between ligand and residue

Pink implies small area close contact surface between (3.0 Å) ligand and residue

Activation barrier corresponds to reaction coordinate within black range; repulsive curve shoulders shown in grey

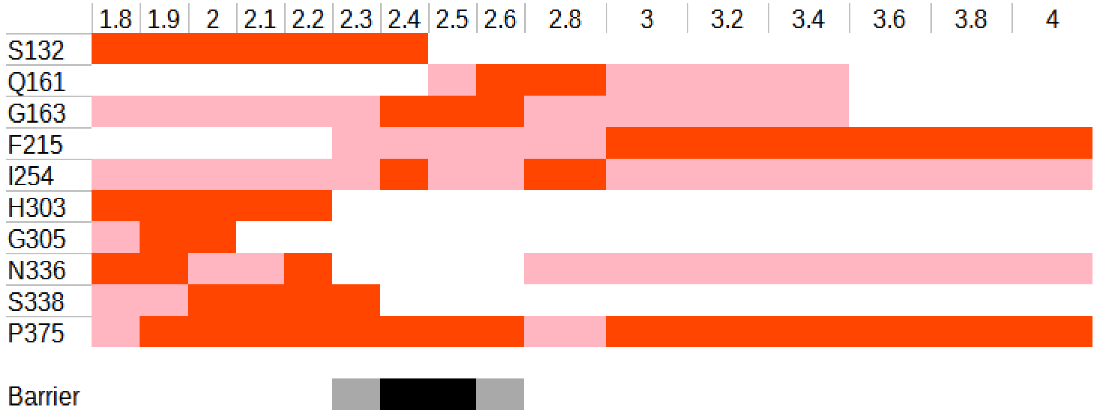

Reaction: cysteine binding to MANT

Notes:

- S132, H303, G305, S338 contacts stabilize product
- Q161, F215 contacts stabilize reactant
- Q161, G163, I254 contacts stabilize transition state
- N336 contacts destabilize transition state

>> Not many great mutation prospects, but S338→T338 may enhance quinolone production at the expense of larger initial substrates.

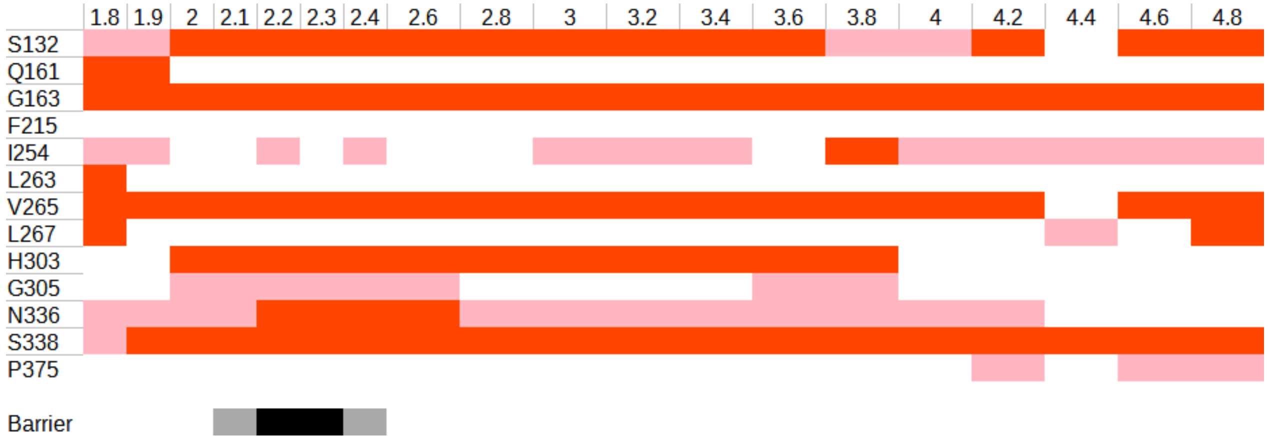

Reaction: First ketide insertion

Notes:

- Q161, L263 contacts stabilize product
- I254, P375 contacts stabilize reactant
- H303, G305 contacts stabilize transition state
- L267 contacts destabilize transition state

>> L267→V267 might improve quinolone & acridone kinetics, but might also reduce enzyme specificity.

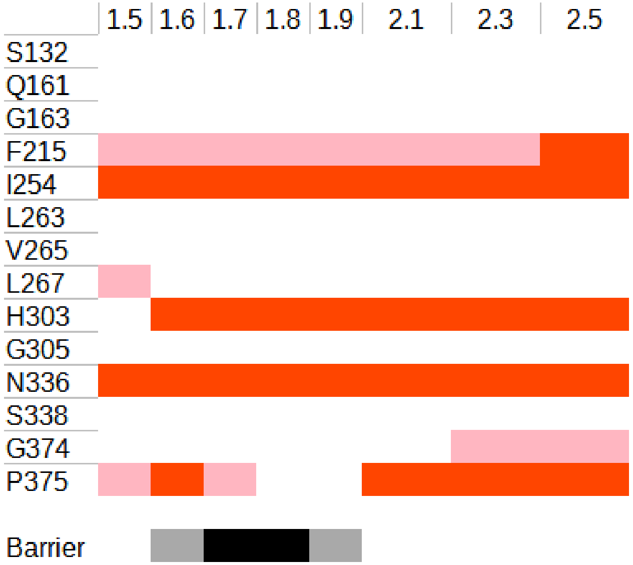

Reaction: Quinolone closure

Notes:

- L267 contacts stabilize product
- F215, G374 contacts stabilize reactant
- I254, H303, N336 contacts <u>may</u> stabilize transition state
- P375 contacts destabilize transition state

>> The diagram alone doesn't say this, but looking closely at the complex structure suggests that N336 is often useful, electrostatically, for steering transition states. It is actually possible that that an N336→S336 mutation might provide better TS-stabilizing H-bonds for MANT-conversion. Again, such a mutation might reduce enzyme specificity.

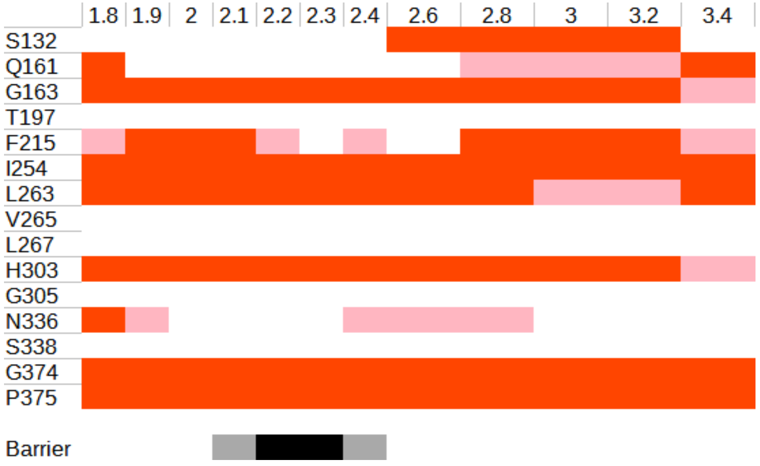

Reaction: Second ketide intermediate

Notes:

- N336 contacts stabilize product
- S132 contacts stabilize reactant
- G163, I254, L263, H303, G374, P375 contacts <u>may</u> stabilize transition state
- Q161, F215, N336 contacts destabilize transition state

>> Q161→V161 and/or F215→V215 might enhance acridone production.
N336→S336, which had been mentioned as potentially benefiting a prior step, may also help this one.

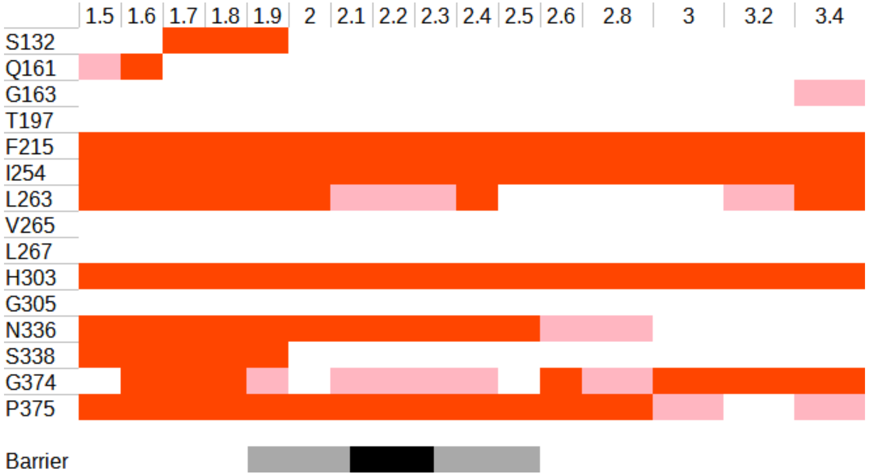

Reaction: Second ketide product

Notes:

- S132, Q161, S338 contacts stabilize product
- G163 contacts <u>may</u> stabilize reactant
- F215, I254, H303, N336, P375 contacts <u>may</u> stabilize transition state
- L263, G374 contacts destabilize transition state

>> S338 is well suited to this reaction; S338→T338 might diminish acridone reaction in favor of quinolone.
Again, mutating Q161 to a similar-sized nonpolar residue (e.g., valine) might improve acridone production.
L263→V263 might also enhance acridone.

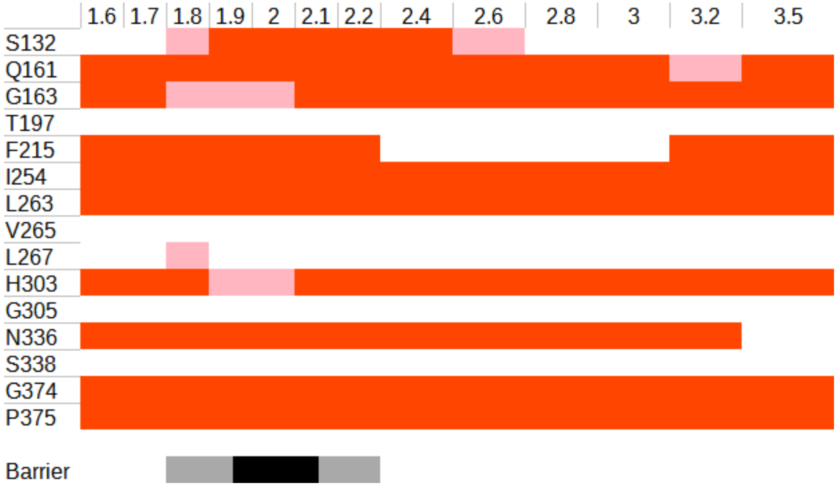

Reaction: Third ketide intermediate

Notes:

- L267 contacts stabilize product
- No clear reactant stabilizers
- S132 contacts stabilize transition state
- G163, H303 contacts destabilize transition state

>> The only residue that might offer a mutational advantage for this acridone formation step is S132 which interacts with the MANT methyl. S132→A132 or S132→V132 might thus help.

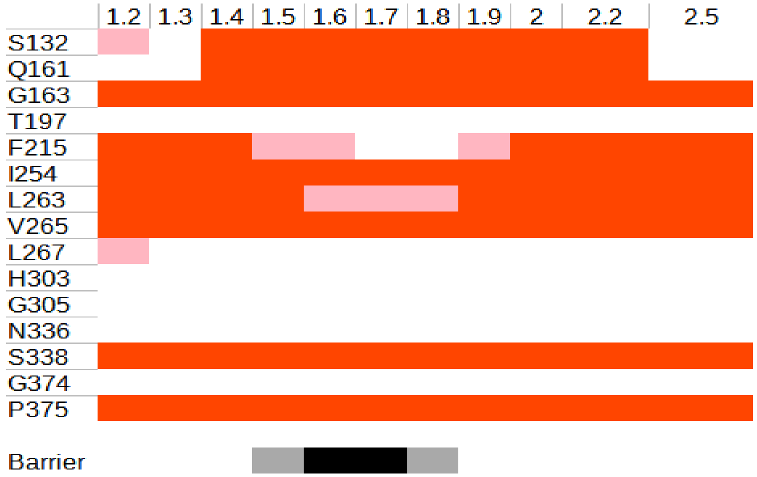

Reaction: Third ketide product

Notes:

- L267 contacts stabilize product
- S132 contacts stabilize reactant
- Q161 contacts stabilize transition state
- F215, L263 contacts destabilize transition state

>> Mutating S132 in the prior step might slow this next step. L267 faces polar end of substrate in a way such that a polar mutation (e.g., L267→Q267) might better stabilize TS and enhance acridone. Finally, L263→V263 might also enhance acridone.

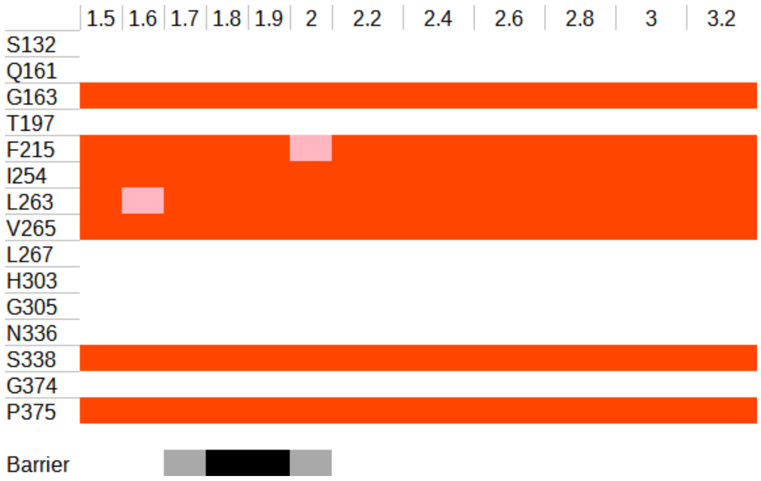

Reaction: macrocycle closure

Notes:

- No clear implications on product stabilization
- No clear implications on reactant stabilization
- G163, I254, V265, S338, P375 contacts <u>may</u> stabilize transition state
- F215, L263 contacts <u>may</u> destabilize transition state

>> F215 pi-stacking might seem crucial for polyketide reactions, but is of unclear importance for quinolone or acridone, so a F215 → V215 mutation might be interesting. Also, this might be another case where L263 → V263 might favor acridone production.

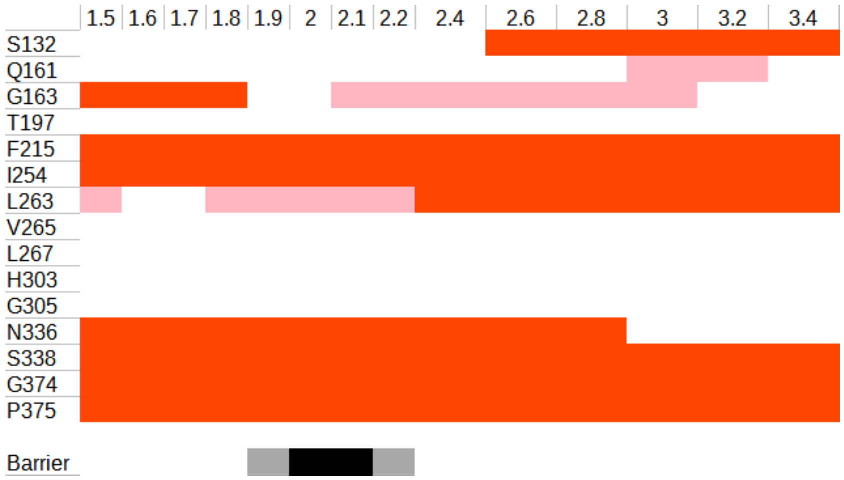

Reaction: cross-macrocycle coupled acridone formation

Notes:

- G163 contacts stabilize product
- S132, Q161, L263 contacts stabilize reactant
- F215, I254, N336, S338, G374, P375 contacts <u>may</u> stabilize transition state
- G163 contacts destabilize transition state

>> Adds interest in S132 → A132 (or S132 → G132) and L263 → V263 mutations for acridone promotion. This is one case where F215 pi stacking may enhance a reaction. N336 → Q336 might enhance this step (contrast with prior favoring smaller S336).

All Supplementary Tables (1-3) are given below

Supplementary Table 1. Crystallization and soaking conditions for native apo, CoASH bound and MANT-CoA substrate bound AmQNS.

	Native apo (6L5U)	CoASH bound (6L7J)	MANT-CoA substrate bound (7CCT)
**Protein concentration (**mg/ml)	10	10	10
**Crystallization Condition**	0.1 HEPES (pH-7.5), 1.4 M sodium citrate tribasic dihydrate	0.1 HEPES (pH-7.5), 1.4 M sodium citrate tribasic dihydrate + CoASH (2mM final concentration)	0.1 HEPES (pH-7.5), 1.4 M sodium citrate tribasic dihydrate
**Additives used (for optimization)**	0.1M magnesium chloride hexahydrate	0.1M $BaCl_2$	0.1M cadmium chloride hydrate
**Drop size**<br>**protein-reservoir ratio**	4 µl drop<br>1:1 ratio	4 µl drop<br>1:1 ratio	4 µl drop<br>1:1 ratio
**Protein concentration (**mg/ml)	10	10	10
**Soaking conditions**			Crystallization Condition + *N*-methylanthraniloyl-CoA (2mM final concentration)
**Soaking time**	-	-	Crystal transferred to 4 µl drop for 30 min
**Cryoprotectant**	20% glycerol	20% glycerol	-

Supplementary Table 2				
**Potential cysteine modification sites on AmQNS** Predictions were done on the server http://pcysmod.omicsbio.info/ using AmQNS sequence (PDB 6L5U), accessed on 14/01/2024 (only significant values are highlighted (those with less fasle positive rate))				
**S-sulfinylation** modification in **Cystein 164** is highlighted in pink				
**Position**	**Modification**	**FPR (False positive rate)**	**Peptide**	**Score**
30	S-palmitoylation	11.64%	STATPPNCVIQADYP	0
60	S-palmitoylation	2.85%	KEKFRRLCEKSMIRK	0
71	S-palmitoylation	0.85%	MIRKRHM**C**LTEEILK	0
84	S-palmitoylation	24.03%	LKANPNMCLHMGTSL	0
130	S-palmitoylation	55.76%	KITHLIFCTSAGVDM	0
164	S-palmitoylation	5.29%	MMIYQQGCYAGATVL	0
190	S-palmitoylation	1.09%	GSRVLIVCSENTVPT	0
341	S-palmitoylation	0.57%	FGNMSSA**C**VLFILDE	0
353	S-palmitoylation	18.71%	LDEMRKTCLEEGKAT	0
30	S-nitrosylation	59.90%	STATPPNCVIQADYP	0.001
60	S-nitrosylation	17.41%	KEKFRRLCEKSMIRK	0.001
71	S-nitrosylation	5.80%	MIRKRHMCLTEEILK	0.002
84	S-nitrosylation	71.21%	LKANPNMCLHMGTSL	0.001
130	S-nitrosylation	0.02%	KITHLIF**C**TSAGVDM	0.246
164	S-nitrosylation	0.01%	MMIYQQG**C**YAGATVL	0.435
190	S-nitrosylation	0.00%	GSRVLIV**C**SENTVPT	4.079
341	S-nitrosylation	13.56%	FGNMSSACVLFILDE	0.001
353	S-nitrosylation	0.00%	LDEMRKT**C**LEEGKAT	1.536
30	S-sulfhydration	68.64%	STATPPNCVIQADYP	0.017
60	S-sulfhydration	51.21%	KEKFRRLCEKSMIRK	0.043
71	S-sulfhydration	23.14%	MIRKRHMCLTEEILK	0.129
84	S-sulfhydration	29.99%	LKANPNMCLHMGTSL	0.099
130	S-sulfhydration	12.93%	KITHLIFCTSAGVDM	0.195
164	S-sulfhydration	32.99%	MMIYQQGCYAGATVL	0.089
190	S-sulfhydration	0.03%	GSRVLIV**C**SENTVPT	0.768
341	S-sulfhydration	30.84%	FGNMSSACVLFILDE	0.097
353	S-sulfhydration	21.95%	LDEMRKTCLEEGKAT	0.135
30	S-sulfenylation	83.35%	STATPPNCVIQADYP	0
60	S-sulfenylation	2.02%	KEKFRRLCEKSMIRK	0.384
71	S-sulfenylation	0.09%	MIRKRHM**C**LTEEILK	0.748
84	S-sulfenylation	58.31%	LKANPNMCLHMGTSL	0
130	S-sulfenylation	19.16%	KITHLIFCTSAGVDM	0.023
164	S-sulfenylation	21.37%	MMIYQQGCYAGATVL	0.016
190	S-sulfenylation	11.92%	GSRVLIVCSENTVPT	0.071
341	S-sulfenylation	25.29%	FGNMSSACVLFILDE	0.008
353	S-sulfenylation	8.64%	LDEMRKTCLEEGKAT	0.12
30	S-sulfinylation	50.53%	STATPPNCVIQADYP	0

60	S-sulfinylation	45.56%	KEKFRRLCEKSMIRK	0
71	S-sulfinylation	52.04%	MIRKRHMCLTEEILK	0
84	S-sulfinylation	49.44%	LKANPNMCLHMGTSL	0
130	S-sulfinylation	3.58%	KITHLIFCTSAGVDM	0.001
164	**S-sulfinylation**	0.02%	MMIYQQG**C**YAGATVL	0.822
190	S-sulfinylation	26.85%	GSRVLIVCSENTVPT	0
341	S-sulfinylation	82.65%	FGNMSSACVLFILDE	0
353	S-sulfinylation	9.93%	LDEMRKTCLEEGKAT	0

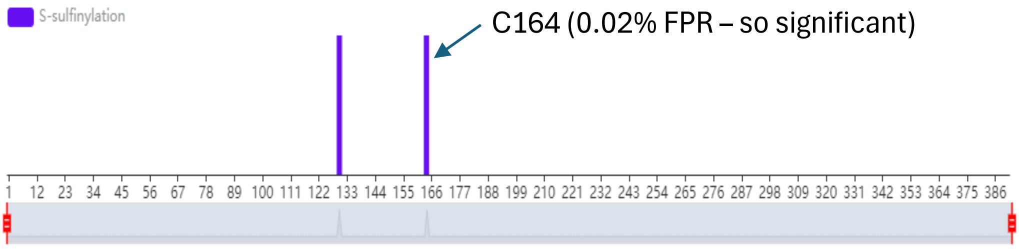

Supplementary Table 3: MANT quinolone and acridone kinetics

Structural dependency for MANT quinolone kinetics and thermodynamics

	1. Mant-CoA binds C164	2. Malonyl-CoA inserts ketide	3. Quinolone closure & product release
MANT	Enthalpy: 36.0 kcal/mol<br>Activation: 57.3 kcal/mol	Enthalpy: -3.5 kcal/mol<br>Activation: 39.6 kcal/mol	Enthalpy: -5.3 kcal/mol<br>Activation: 15.1 kcal/mol
MANT-pyridine	Enthalpy: 40.4 kcal/mol<br>Activation: 60.2 kcal/mol	Enthalpy: -9.2 kcal/mol<br>Activation: 31.4 kcal/mol	Enthalpy -4.1 kcal/mol<br>Activation: 23.0 kcal/mol
MANT-chloro	Enthalpy: 32.7 kcal/mol<br>Activation: 53.5 kcal/mol	Enthalpy: 4.4 kcal/mol<br>Activation: 44.1 kcal/mol	Enthalpy: -4.6 kcal/mol<br>Activation: 19.9 kcal/mol

Structural dependency for MANT acridone kinetics and thermodynamics

	1. 2nd Malonyl-CoA inserts ketide	2. 3rd Malonyl-CoA inserts ketide	3. Acridone closure and product release
	Enthalpy: 10.4 kcal/mol<br>Activation: 59.9 kcal/mol	Enthalpy: 9.4 kcal/mol<br>Activation: 53 kcal/mol	Enthalpy: -16.0 kcal/mol<br>Activation: 38.3 kcal/mol
