# Supplementary material for: Structural and mechanistic insights into Quinolone Synthase to address its functional promiscuity
Source: Commun Biol. 2024 May 14;7:566. doi: 10.1038/s42003-024-06152-2 (PMC11093982; doi:10.1038/s42003-024-06152-2)
Supplement: Supplementary file 2 — Description of Additional Supplementary Files [file 42003_2024_6152_MOESM2_ESM.pdf]

Description of Additional Supplementary Files

File name: Supplementary Data 1
Description: Data used for Homolog screening.

File name: Supplementary Data 2
Description: Rutacean homologs.

File name: Supplementary Data 3
Description: Other homologs.

File name: Supplementary Data 4
Description: Other homologs.

File name: Supplementary Movie 1
Description: Transition steps of AmQNS catalysis.

File name: Supplementary Movie 2
Description: Transition steps of AmQNS catalysis.

File name: Supplementary Movie 3
Description: Transition steps of AmQNS catalysis.
